# Adolescent and caregiver preferences for juvenile idiopathic arthritis treatment: a discrete-choice experiment

**DOI:** 10.1186/s12969-023-00906-8

**Published:** 2023-10-21

**Authors:** Flora McErlane, Marco Boeri, Cooper Bussberg, Joseph C. Cappelleri, Rebecca Germino, Lori Stockert, Caroline Vass, Adam M. Huber

**Affiliations:** 1 Paediatric Rheumatology Department, Great North Children’s Hospital, Newcastle Upon Tyne, UK; 2 RTI Health Solutions, Belfast, Northern Ireland UK; 3 Queen’s University Belfast, Belfast, Northern Ireland UK; 4 Patient-Centered Outcomes, OPEN Health, Belfast, UK; 5 RTI Health Solutions, Research Triangle Park, NC USA; 6 Pfizer, Groton, CT USA; 7 Pfizer, New York, NY USA; 8 Pfizer, Collegeville, PA USA; 9 RTI Health Solutions, Manchester, UK; 10 The University of Manchester, Manchester, UK; 11 Division of Pediatric Rheumatology, IWK Health Centre, Dalhousie University, Halifax, NS Canada

**Keywords:** Caregiver, Patient, Juvenile idiopathic arthritis, Stated preferences

## Abstract

**Background:**

This study aimed to elicit and quantify preferences for treatments for juvenile idiopathic arthritis (JIA).

**Methods:**

We conducted a discrete-choice experiment among adolescents with JIA in the United States (US) (*n* = 197) and United Kingdom (UK) (*n* = 100) and caregivers of children with JIA in the US (*n* = 207) and UK (*n* = 200). In a series of questions, respondents chose between experimentally designed profiles for hypothetical JIA treatments that varied in efficacy (symptom control; time until next flare-up), side effects (stomachache, nausea, and vomiting; headaches), mode and frequency of administration, and the need for combination therapy. Using a random-parameters logit model, we estimated preference weights for these attributes, from which we derived their conditional relative importance.

**Results:**

On average, respondents preferred greater symptom control; greater time until the next flare-up; less stomachache, nausea, and vomiting; and fewer headaches. However, adolescents and caregivers in the US were generally indifferent across varying modes and frequencies of administration. UK adolescents and caregivers preferred tablets, syrup, or injections to intravenous infusions. US and UK adolescents were indifferent between treatment with monotherapy or combination therapy; caregivers in the UK preferred treatment with combination therapy to monotherapy. Subgroup analysis showed preference heterogeneity across characteristics including gender, treatment experience, and symptom experience in both adolescents and caregivers.

**Conclusions:**

Improved symptom control, prolonged time to next flare-up, and avoidance of adverse events such as headache, stomachache, nausea, and vomiting are desirable characteristics of treatment regimens for adolescents with JIA and their caregivers.

**Supplementary Information:**

The online version contains supplementary material available at 10.1186/s12969-023-00906-8.

## Background

Juvenile idiopathic arthritis (JIA) is the most common paediatric rheumatic disease and a leading cause of childhood disability [1]. Treatment for JIA primarily consists of nonsteroidal anti-inflammatory drugs, rescue corticosteroid regimens, conventional synthetic disease-modifying antirheumatic drugs (csDMARDs), biologics, and targeted synthetic (ts)DMARDs (e.g., Janus kinase [JAK] inhibitors) to reduce inflammation, manage pain, and/or regulate the immune system. JIA and its treatments can significantly impair patients’ health-related quality of life [2], with challenging treatment experiences including potential side effects and the need for frequent injections.

Although several studies have investigated preferences for treatments of common chronic immune and inflammatory diseases [3–6], few studies have explored the preferences of young people or their caregivers in relation to the management or treatment of JIA. Burnett and colleagues [7] elicited preferences from parents of children with JIA to understand how they traded off treatment attributes and health outcomes; these data were later analysed to estimate willingness to pay for biologic treatments [8]. Another study quantified parents’ preferences for the management of foot problems in JIA [9]. Formative qualitative research also has been conducted with paediatric rheumatologists to understand their decision-making process when treating JIA patients with biologics, in order to inform attribute selection for a future best–worst scaling survey [10]. Heretofore, however, most studies have focused on understanding how parents and guardians make choices about treatment rather than eliciting preferences from patients with JIA.

A treatment approach that considers patients’ and caregivers’ preferences and priorities is critical in JIA. To inform a patient-centric care framework for JIA, the objectives of this study were to elicit adolescent and caregiver preferences in relation to efficacy, safety, and administration attributes of JIA treatment in the US and United Kingdom (UK) and to quantify the relative importance of these treatment attributes.

## Methods

### Study design

For this study, a cross-sectional, web-based discrete-choice experiment (DCE) survey was developed and administered to adolescent and caregiver participants in the US and UK between May and August 2021. The study design and analyses were developed following good research practices as defined by ISPOR guidelines and standard practice in preference studies [11–13]. The study was reviewed and deemed exempt from full review by the institutional review board of RTI International, a nonprofit research organisation. All adult (caregiver) survey respondents provided informed consent before completing the study. All adolescent participants also provided their assent to participate in the study, along with the consent of their parent or legal guardian. The DCE method was chosen to quantify preferences for different attributes of JIA treatments.

### Study population

A purposive sample of potentially eligible adolescent and caregiver respondents was identified by Global Perspectives through patient databases, physician referrals, social media, patient associations, and Global Perspectives’ online panels; members of this sample were invited to be screened for study eligibility. Adolescent respondents (aged 14–17 years) with a self-reported physician diagnosis of JIA who were able to read and understand English and provide online informed consent were recruited in the US and UK. Caregiver respondents who were (1) the parent or guardian (aged 18 years or older) of a child or adolescent (aged younger than 18) with a self-reported physician diagnosis of JIA and (2) able to read and understand English and provide online informed consent were recruited in the US and UK. Respondents were compensated for their time in completing the survey ($50 for US respondents and £30 for UK respondents).

### Survey instrument

**Fig. 1:**
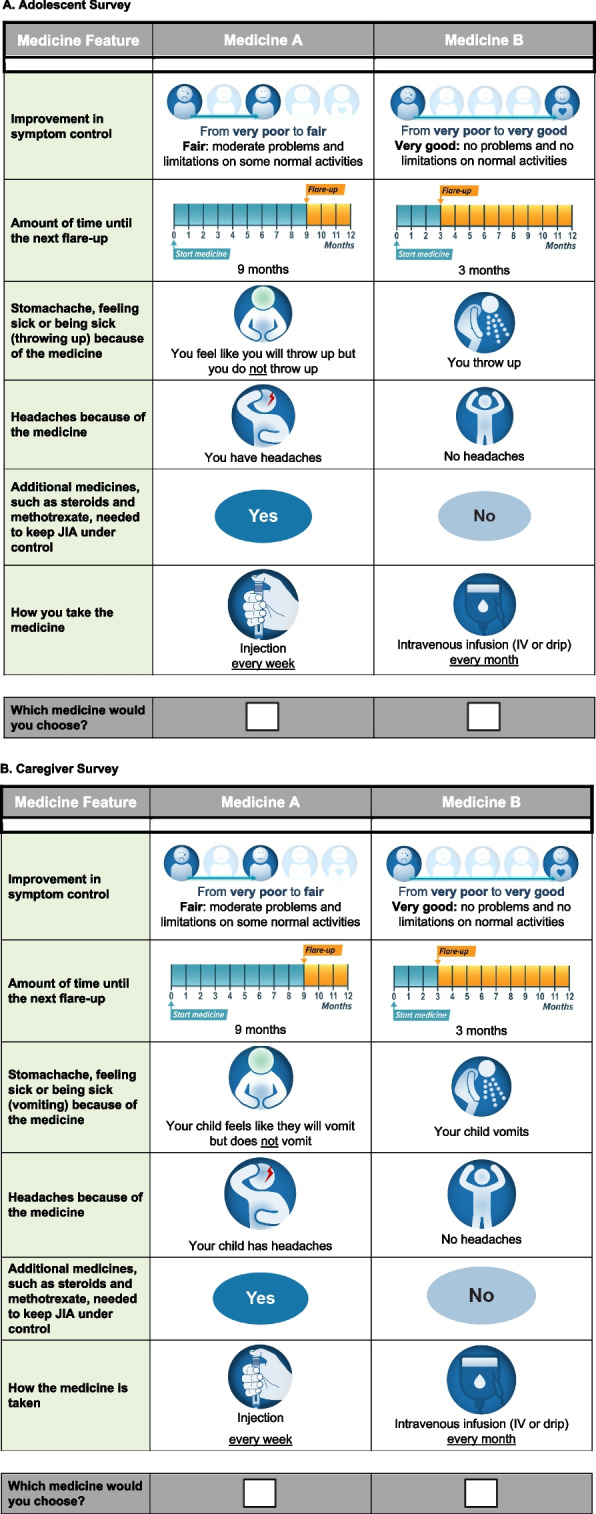
Example of a Discrete-Choice Experiment Question

In the DCE, respondents were presented with a series of 12 choice questions that each asked them to choose between experimentally designed hypothetical JIA treatment profiles. A sample choice question is shown in Fig. 1. The hypothetical treatment profiles varied along 6 attributes that are important to individuals with JIA and their caregivers and that differentiate the available treatment options: (1) symptom control; (2) time until next flare-up; (3) stomachache, nausea, and vomiting; (4) headache; (5) need for combination therapy (methotrexate or steroids, or both); and (6) mode and frequency of administration (Table 1). The treatment attributes and levels were selected based on a review of clinical trial data for JIA treatments and on other information from previous preference studies in JIA and of applications of a JAK inhibitor [1, 7, 9, 14]. Rowen et al. (2020) highlight the importance of an appropriate selection of tasks, design, framing, and presentation in studies that use DCEs to elicit preferences from adolescents [15]. It often is cognitively challenging for respondents (particularly adolescent patients) to complete choice tasks with many probabilistic attributes. In this study, the information in both caregiver and adolescent samples was presented using simple language that was appropriate for the reading level expected of the youngest respondents.

**Table 1:** Attributes and levels for the discrete-choice experiment

Technical attribute label	Caregiver/adolescent-facing attribute label	Caregiver-facing attribute levels	Adolescent-facing attribute levels
Symptom control ^a^	Improvement in symptom control	From very poor to poor	From very poor to poor
		From very poor to fair	From very poor to fair
		From very poor to good	From very poor to good
		From very poor to very good	From very poor to very good
Time until next flare-up ^b^	Amount of time until the next flare-up	1 month	1 month
		3 months	3 months
		5 months	5 months
		9 months	9 months
Stomachache, nausea, and vomiting ^c^	Stomachache, feeling sick or being sick (throwing up/vomiting) because of the medicine	None	None
		Your child has a stomachache but does not feel like vomiting	Tummy pain (but you do not feel like you will throw up)
		Your child feels like vomiting but does not vomit	You feel like you will throw up, but you do not throw up
		Your child vomits	You throw up
Headache	Headaches because of the medicine	No headaches	No headaches
		Your child has headaches	You have headaches
Need for combination therapy (methotrexate or steroids, or both)^d^	Additional medicines, such as steroids and methotrexate, needed to keep JIA under control	No	No
		Yes	Yes
Mode and frequency of administration ^e^	How the medicine is taken	Tablets or liquid/syrup ^f^ twice a day	Tablets or liquid twice a day
		Injection every week	Injection every week
		Injection every 2 weeks	Injection every 2 weeks
		IV infusion (IV or drip) every month	IV infusion (IV or drip) every month

*IV* Intravenous, *JIA* Juvenile idiopathic arthritis. Note: The survey instrument for adults used slightly different language than the survey instrument for adolescents

^a^Levels for this attribute were developed based on the findings from Davies et al. [16]

^b^Levels for this attribute were developed based on the findings from Ruperto et al. [17]

^c^Levels for this attribute were developed based on the findings from Brunner et al. [14]

^d^Levels for this attribute were developed based on the findings from Fleischmann et al. [18]

^e^Levels for this attribute were developed based on the findings from Ruperto et al. [17]

^f^The United States survey labelled this attribute level as “tablets or liquid twice a day” and the United Kingdom survey labelled this attribute level as “tablets or syrup twice a day”

The survey instrument was pretested in semistructured online interviews via teleconference with a convenience sample of 18 adolescents (9 in the US and 9 in the UK) and 20 caregivers (10 in the US and 10 in the UK) to test the comprehensibility of the DCE survey instrument, the relevance and comprehensiveness of the attributes, the appropriateness of descriptive information, and the level of difficulty of the DCE questions for the target population. Each individual interview was conducted via telephone or videoconference by 2 experienced interviewers. The pretests initially included adolescent interviewees as young as 12 years. While older participants (aged ≥ 14 years) generally understood the DCE exercise, 12-year-old participants and some 13-year-old participants struggled to answer the survey questions. Therefore, for the final survey, the minimum age of eligible adolescents was adjusted from 12 to 14 years.

### Statistical analysis

The data from the DCE were analysed using a random-parameters logit (RPL) model, following good research practice guidelines [12, 19–22]. RPL models relate treatment choices from each respondent to the attribute levels of each treatment profile in the choice questions, yielding preference-weight estimates for the attributes and levels included in the DCE. By estimating a distribution around each mean preference parameter, RPL models mitigate potential estimation bias in the mean preference-weight estimates that may occur because of unobserved preference heterogeneity among respondents [21, 22]. Preference weights estimated from an RPL model indicate the relative strength of preference for each attribute level included in the survey; more-preferred outcomes have higher preference weights.

These preference weights were used to calculate the importance of each attribute, conditional on the range of levels considered and relative to all other attributes included in the survey [12, 23]. The conditional relative importance (CRI) of each attribute was calculated as the difference in preference weights for the most-preferred and least-preferred level of that attribute. The results were rescaled so that all CRI estimates summed to 100 and each CRI estimate was a proportion of 100.

Finally, although the RPL model controls for unobserved heterogeneity in preferences, it does not identify observable characteristics that may be associated with differences in preferences. However, it is possible to explore observed preference heterogeneity using subgroup analysis [24]. Such analysis was conducted in this study for prespecified, mutually exclusive subgroups defined by age, gender, treatment experience, and symptom experience.

## Results

### Respondent characteristics

In the US, the survey instrument was completed by 197 adolescent patients (of 235 invited to be screened for the survey, including 82 invited through email and online panel portals and 153 through referral from caregivers) and 207 caregivers (of 4,309 who accessed the survey and completed the screening questions; 556 of these individuals met the eligibility criteria and consented to participate). In the UK, the survey instrument was completed by 100 adolescent patients (of 120 invited to be screened for the survey, all recruited through referral from caregivers) and 200 caregivers (of 4,382 who accessed the survey and completed the screening questions) (Table 2). Among the 197 US adolescents, 117 identified as male (59.4%) and 154 identified as White (78.2%); the average age was approximately 15 years (range, 14–17 years). Among the 207 US caregivers, 145 identified as male (70.0%) and 163 identified as White (78.7%). The average age was approximately 38 years (range, 21–62 years), and the average age of the caregiver’s child was 12 years (range, 0–17 years). Among the 100 UK adolescents, 80 identified as male (80.0%) and 89 identified as White (89.0%); the average age was approximately 15 years (range, 14–17 years). Among the 200 UK caregivers, 144 identified as male (72.0%) and 167 identified as White (83.5%). The average age was approximately 39 years (range, 21–64 years), and the average age of the caregiver’s child was 12 years (range, 2–17 years).

**Table 2:** Demographic characteristics of the respondents

Question	US		UK	
	**Adolescents (*N* = 197) n (%)**	**Caregivers (*N* = 207) n (%)**	**Adolescents (*N* = 100) n (%)**	**Caregivers (*N* = 200) n (%)**
How old are you?				
Mean (SD)	15.2 (0.9)	38.1 (6.5)	14.9 (0.7)	38.8 (6.9)
How old is your child with JIA?				
Mean (SD)	N/A	11.7 (4.0)	N/A	11.9 (3.2)
Do you consider yourself to be?				
Female	78 (39.6%)	60 (29.0%)	20 (20.0%)	56 (28.0%)
Male	117 (59.4%)	145 (70.0%)	80 (80.0%)	144 (72.0%)
Transgender	0 (0.0%)	0 (0.0%)	0 (0.0%)	0 (0.0%)
Other (e.g., gender fluid, non-binary)	1 (0.5%)	1 (0.5%)	0 (0.0%)	0 (0.0%)
Prefer not to answer	1 (0.5%)	1 (0.5%)	0 (0.0%)	0 (0.0%)
What is your child’s gender?				
Female	N/A	60 (29.0%)	N/A	69 (34.5%)
Male	N/A	145 (70.0%)	N/A	131 (65.5%)
Transgender	N/A	1 (0.5%)	N/A	0 (0.0%)
Other (e.g., gender fluid, non-binary)	N/A	0 (0.0%)	N/A	0 (0.0%)
Prefer not to answer	N/A	1 (0.5%)	N/A	0 (0.0%)
What is your ethnicity? (Please select all that apply) ^a^				
American Indian or Alaska Native	1 (0.5%)	4 (1.9%)	N/A	N/A
Black or African American, non-Hispanic	29 (14.7%)	17 (8.2%)	N/A	N/A
Black/African/Caribbean/Black British	N/A	N/A	3 (3.0%)	11 (5.5%)
Asian or Pacific Islander	1 (0.5%)	9 (4.3%)	N/A	N/A
Asian/Asian British	N/A	N/A	9 (9.0%)	26 (13.0%)
White, non-Hispanic	154 (78.2%)	163 (78.7%)	89 (89.0%)	167 (83.5%)
Hispanic	15 (7.6%)	18 (8.7%)	N/A	N/A
Other/other ethnic groups	0 (0.0%)	0 (0.0%)	0 (0.0%)	0 (0.0%)
Mixed/multiple ethnic groups	N/A	N/A	1 (1.0%)	5 (2.5%)
Prefer not to share	0 (0.0%)	1 (0.5%)	0 (0.0%)	0 (0.0%)
What is your child’s ethnicity? (Please select all that apply) ^a^				
American Indian or Alaska Native	N/A	5 (2.4%)	N/A	N/A
Black or African American, non-Hispanic	N/A	17 (8.2%)	N/A	N/A
Black/African/Caribbean/Black British	N/A	N/A	N/A	11 (5.5%)
Asian or Pacific Islander	N/A	8 (3.9%)	N/A	N/A
Asian/Asian British	N/A	N/A	N/A	28 (14.0%)
White, non-Hispanic	N/A	163 (78.7%)	N/A	166 (83.0%)
Hispanic	N/A	19 (9.2%)	N/A	N/A
Other/other ethnic groups	N/A	0 (0.0%)	N/A	0 (0.0%)
Prefer not to share	N/A	1 (0.5%)	N/A	0 (0.0%)
Mixed/multiple ethnic groups	N/A	N/A	N/A	4 (2.0%)

*N/A* Not applicable

^a^The total may exceed 100.0% because respondents could select multiple responses to this question

Percentages are provided to 1 decimal place and as such, rounding may mean totals do not equal 100.0%

### Preference weights and conditional relative importance of attributes

**Fig. 2:**
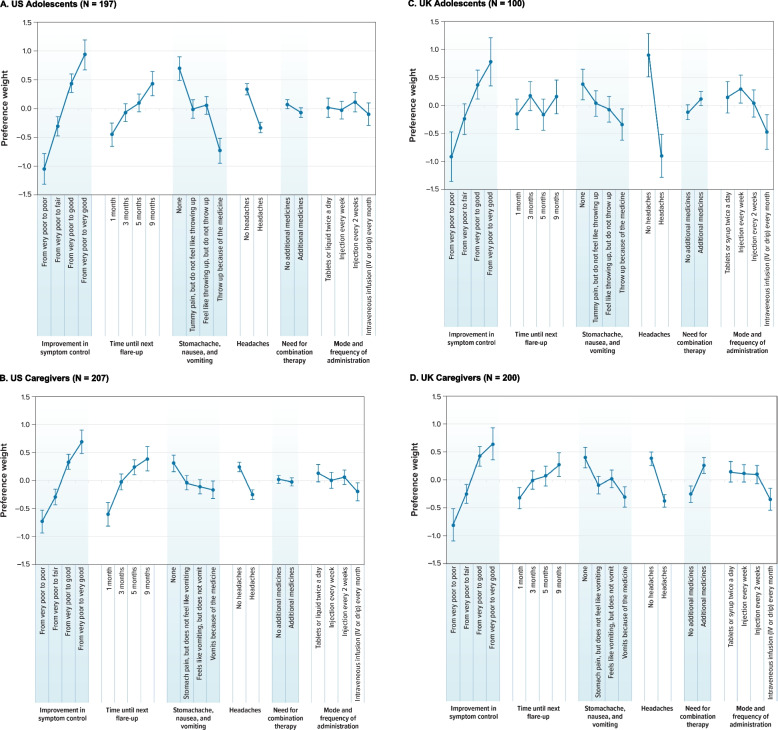
Random-Parameters Logit Model Estimates: Preference Weights. **A** US Adolescents (*N* = 197). **B** US Caregivers (*N* = 207). **C** UK Adolescents (*N* = 100). **D** UK Caregivers (*N* = 200). *CI* Confidence interval, *DCE* Discrete-choice experiment, *IV* Intravenous. Note: Attributes are presented in the order in which they appeared in the DCE questions. The vertical bars around each mean preference weight represent the 95% CI around the point estimate. Because all attribute levels are effects coded, the sum of preference weights across levels of an attribute equals 0. Within each attribute, a higher preference weight indicates that a level is more preferred. The change in utility associated with a change in the levels of each attribute is represented by the vertical distance between the preference weights for any 2 levels of that attribute. Larger differences between preference weights indicate that respondents viewed the change as having a relatively greater effect on overall utility. For example, looking at Fig. 2A, a change in improvement in symptom control from “very poor to poor” to “very poor to fair” yields a utility increase of 0.745 (− 0.310 − [− 1.054]), whereas an increase in the time until next flare-up from 1 to 5 months yields a smaller utility increase of 0.547 (− 0.091 − [− 0.456]). Although both changes yield positive utility increases, the change from “very poor to poor” to “very poor to fair” yields a change 1.36 times more important than the change from 1 month until next flare-up to 5 months until next flare-up (0.745 ÷ 0.547). Alternatively, a change from no stomachache, nausea, or vomiting to stomachache (but with no feelings of throwing up) yields a negative utility change of − 0.703 (− 0.011 − 0.692), whereas a change from no headaches caused by the medicine to having headaches caused from the medicine yields a negative utility change of − 0.666 (− 0.333 − 0.333)

**Fig. 3:**
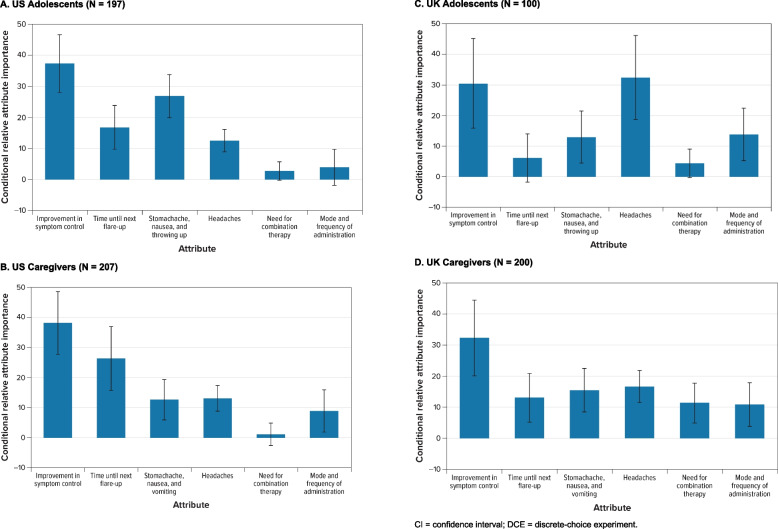
Random-Parameters Logit Model Estimates: Scaled Conditional Relative Attribute Importance. **A** US Adolescents (*N* = 197). **B** US Caregivers (*N* = 207). **C** UK Adolescents (*N* = 100). **D** UK Caregivers (*N* = 200). *CI* Confidence interval, *DCE* Discrete-choice experiment. Note: Attributes are presented in the order in which they appeared in the DCE questions. For each attribute, the conditional relative importance was computed as the difference between the preference weights on the most and the least preferred level. Once computed, the conditional relative importance estimates were rescaled so that their sum was equal to 100; therefore, each one can be interpreted as the proportion of utility that can be gained by improving one attribute from the least to the most preferred level relative to the maximum utility gained from improving all attributes from the least to the most preferred level. The standard errors and the 95% CI for the differences were calculated using the delta method. The 95% CI around the point estimate is represented by the black vertical bars on top of the blue bars. For example, looking at Fig. 3A, the largest CRI is improvement in symptom control, followed by stomachache, nausea, and throwing up; time until next flare-up; headaches; mode and frequency of administration; and need for combination therapy

Figure 2 plots the mean preference-weight estimate for each attribute level for the 4 cohorts. The vertical bars around each preference weight represent the 95% confidence interval. Preference weights are relative to each other and do not have an absolute interpretation. The attribute levels with larger preference weights are preferred to attribute levels with smaller preference weights. Figure 3 displays the CRI for each attribute for the 4 cohorts.

### US respondents

As shown in Fig. 2A and B, the preference weights for US adolescents and US caregivers were ordered as expected, with better outcomes being preferred to worse outcomes. On average, US adolescents and caregivers preferred better symptom control; greater time until the next flare-up; less stomachache, nausea, and vomiting; and fewer headaches. US adolescents and caregivers also were indifferent between a treatment with combination therapy and one without, and were generally indifferent across modes of administration, as can be seen by the lack of statistically significant differences between levels.

Among US adolescents, the largest CRI was improvement in symptom control, followed by stomachache, nausea, and vomiting; time until next flare-up; headaches; mode and frequency of administration; and need for combination therapy (Fig. 3A). The CRIs that were not statistically different from each other at the 95% confidence level were time until next flare-up and headaches (*P* = 0.270) and need for combination therapy and mode and frequency of administration (*P* = 0.703).

Among US caregivers, the largest CRI was improvement in symptom control, followed by time until next flare-up; headaches; stomachache, nausea, and vomiting; mode and frequency of administration; and need for combination therapies (Fig. 3B). However, the CRIs for improvement in symptom control and time until next flare-up were not statistically different from each other at the 95% confidence level (*P* = 0.106). Additionally, the CRIs for stomachache, nausea, and vomiting; headaches; and mode and frequency of administration were not statistically different from each other at the 95% confidence level.

### UK respondents

As shown in Figs. 2C and 2D, the preference weights for UK adolescents and caregivers were ordered as expected, with better outcomes being preferred to worse outcomes. On average, adolescents and caregivers preferred better symptom control; less stomachache, nausea, and vomiting; and fewer headaches. They also preferred tablets/syrup and injections to intravenous (IV) infusions.

Among UK adolescents, although the difference in preference weights for a treatment with and without combination therapy was not statistically significant, adolescents appeared to prefer combination therapy (in which they received additional medicines, as opposed to no additional medicines) (Fig. 2C). Generally, there were few statistically significant differences across attribute levels, potentially due to sample size constraints. For example, there were not statistically significantly different preferences across the levels of time until next flare-up, and adolescents in the UK did not have statistically significantly different preferences between treatments by tablets and treatments by injections. Among UK adolescents, the most important attribute was headaches, followed by improvement in symptom control (Fig. 3C). However, the CRIs for headaches and improvement in symptom control were not statistically significantly different from each other (*P* = 0.819). Mode and frequency of administration and stomachache, nausea, and vomiting came next in order of importance. Time until next flare-up and the need for combination therapy were the 2 least important attributes, and the respective CRIs were not statistically significantly different from 0.

Like UK adolescents, UK caregivers preferred treatments that require combination therapy to those that do not require combination therapy (Fig. 2D). As with the UK adolescent sample, this could possibly be explained by previous experience with combination therapy or because the respondents interpreted having to take an additional medicine as an indicator of more efficacy. Among UK caregivers, the largest CRI was for symptom control, followed by headaches; stomachache, nausea, and vomiting; time until next flare-up; need for combination therapy; and mode and frequency of administration (Fig. 3D). The CRI for improvement in symptom control was statistically significantly different from the CRIs for all other attributes at the 95% confidence level. However, none of the CRIs were statistically significantly different from each other at the 95% confidence level for the other 5 attributes: time until next flare-up; stomachache, nausea, and vomiting; headaches; need for combination therapy; and mode and frequency of administration.

### Subgroup analysis

Results of the subgroup analyses for the US cohorts revealed significant preference heterogeneity for adolescents across multiple sample characteristics but minimal preference heterogeneity for caregivers (Table 3 and Figures S1-S17, Supplemental Appendix A). Systematically different preferences were found in the US adolescent cohort among subgroups defined by gender, methotrexate experience, headache experience, and stomachache experience and in the US adult cohort only among the subgroup defined by the caregiver’s child’s experience with vomiting.

**Table 3:** Subgroup analysis, by cohort

Subgroup set and sample size (n)	Summary of the results
**US adolescents, *N* = 197**	
**Age**^**a**^	Preferences were not statistically different between younger and older adolescents in the study
Younger than median age (*n* = 46)	
At or older than median age (*n* = 151)	
*P* *value* = *0.738*	
**Gender**	Gender was a significant driver of preferences. Improvement in symptom control was by far the most important attribute for respondents who did not identify as female, while respondents who identified as female generally placed similar importance on (1) symptom control; (2) time until the next flare-up; and (3) avoiding stomachaches, nausea, and vomiting. In addition, respondents who identified as female significantly disliked getting the treatment by IV infusion every month and preferred other modes and frequencies of administration; they also significantly preferred having no additional medicines
Does not identify as female (*n* = 119)	
Identifies as female (*n* = 78)	
*P* *value* = *0.004*	
**Experience with methotrexate**	Experience with methotrexate was also a significant driver of preferences
Has no experience with methotrexate (*n* = 124)	Improvement in symptom control was by far the most important attribute for respondents with methotrexate experience. Respondents without methotrexate experience placed similar importance on improvement in symptom control and avoiding stomachaches, nausea, and vomiting. Preferences for mode and for combination therapy were different across these 2 groups
Has experience with methotrexate (*n* = 73)	
* P* *value* = *0.003*	
**Experience with biologics**	Preferences were not statistically different between adolescents with and without experience with biologics, although respondents with biologics experience placed more importance on improvement in symptom control, relatively to other attributes
Has no experience with biologics (n = 59)	
Has experience with biologics (n = 138)	
*P* *value* = *0.307*	
**Experience with injections**	Preferences were not statistically different between adolescents with and without experience with injections, mainly due to the small sample size (and resulting wide confidence intervals). However, respondents with injection experience considered improvement in symptom control the most important attribute, which was not the case for respondents without injection experience
Has no experience with injections (*n* = 43)	
Has experience with injections (*n* = 154)	
*P* *value* = *0.239*	
**Experience with headaches**	Headache experience was a significant driver of preference heterogeneity. Improvement in symptom control was the most important attribute for respondents with headache experience, while to respondents without headache experience, efficacy and adverse event attributes generally had similar importance
Has no experience with headaches (*n* = 34)	
Has experience with headaches (*n* = 163)	
*P* *value* = *0.032*	
**Experience with stomachaches**	Experience with stomachache was a significant driver of preference heterogeneity as well. Improvement in symptom control was the most important attribute for respondents with stomachache experience, while respondents without stomachache experience generally considered (1) avoiding stomachaches, nausea, and vomiting and (2) time until next flare-up as most important. Respondents with no stomachache experience strongly disliked IV infusion compared to other modes of administration and preferred no additional medicine
Has no experience with stomachaches (*n* = 22)	
Has experience with stomachaches (*n* = 175)	
*P* *value* = *0.024*	
**Experience with vomiting**	Preferences were not statistically different between adolescents with and without experience vomiting, although respondents with vomiting experience considered improvement in symptom control the most important attribute, while those without vomiting experience placed similar importance on efficacy and on avoiding stomachaches, nausea, and vomiting
Has no experience with vomiting (*n* = 51)	
Has experience with vomiting (*n* = 146)	
*P* *value* = *0.513*	
**US caregivers, *N* = 207**	
**Child’s median age**^**b**^	Preferences were not systematically statistically different between caregivers with younger and older children, although respondents with children at median age or older preferred treatments that delayed the time until next flare-up
Child is younger than median age (*n* = 100)	
Child is at or older than median age (*n* = 107)	
*P* *value* = *0.289*	
**Child’s gender**	Child’s gender was not a driver of preference heterogeneity overall; however, caregivers with a child who did not identify as female preferred no additional medicines, while caregivers with a child who identified as female preferred treatments that required additional medicines
Child does not identify as female (*n* = 147)	
Child identifies as female (*n* = 60)	
* P* *value* = *0.870*	
**Caregiver’s level of education**	Although preferences between these 2 groups were not systematically different at the 95% level of confidence, improvement in symptom control was the most important attribute for caregivers with a 4-year degree or higher, while caregivers with less than a 4-year degree placed the most importance on increasing the time until the next flare-up. Confidence intervals were very large for this group
Caregiver has less than a 4-year degree (*n* = 47)	
Caregiver has a 4-year degree or higher (*n* = 160)	
*P* *value* = *0.195*	
**Caregiver’s child has experience with methotrexate**	Preferences were not systematically statistically different between caregivers with a child who has experience with methotrexate and those with a child with no experience with methotrexate
Child has no experience with methotrexate (*n* = 120)	
Child has experience with methotrexate (*n* = 87)	
*P* *value* = *0.801*	
**Caregiver’s child has experience with biologics**	Although the child’s experience with biologics was not a driver of preference heterogeneity among caregivers, caregivers with a child who has experience with biologics considered improvement in symptom control the most important attribute. Additionally, these respondents did not have systematically different preferences across treatments that increase the time until next flare-up by 3, 5, or 9 months but did prefer these levels to a treatment that only increases the time until next flare-up by 1 month
Child has no experience with biologics (*n* = 76)	
Child has experience with biologics (*n* = 131)	
*P* *value* = *0.248*	
**Caregiver’s child has experience with injections**	Although preferences were not systematically different between these 2 groups at the 95% level of confidence, caregivers with a child who has no experience with injections considered improvement in symptom control the most important attribute (although confidence intervals were very large), while caregivers with a child who has experience with injections considered improvement in symptom control and time until the next flare-up the most important attributes in the study
Child has no experience with injections (*n* = 59)	
Child has experience with injections (*n* = 148)	
*P* *value* = *0.115*	
**Caregiver’s child has experience with headaches**	Preferences were similar across this subgroup set, although caregivers with a child who has not experienced headaches considered increasing the time until the next flare-up and improvement in symptom control the most important attributes and strongly disliked injections every 2 weeks, while caregivers with a child who has experience with headaches placed the most importance on improvements in symptom control and strongly disliked IV infusion every month
Child has no experience with headaches (*n* = 59)	
Child has experience with headaches (*n* = 148)	
*P* *value* = *0.095*	
**Caregiver’s child has experience with stomachaches**	Preferences across these 2 groups were not systematically different; however, time until the next flare-up and improvement in symptom control were the most important attributes for caregivers with a child who has not experienced stomachaches, while improvement in symptom control was the most important attribute for respondents with a child who has experienced stomachaches. In addition, preferences for mode and frequency of administration were different across the 2 groups
Child has no experience with stomachaches (*n* = 47)	
Child has experience with stomachaches (*n* = 160)	
* P* *value* = *0.262*	
**Caregiver’s child has experience with vomiting**	The child’s experience with vomiting significantly drove preference heterogeneity. Caregivers with a child with no experience with vomiting placed most importance on improvement in symptom control and increasing the time until the next flare-up. These respondents preferred a treatment by oral tablet or liquid to a treatment by injection every week, but were indifferent between tablets, injection every 2 weeks, or IV infusion every month. Improvement in symptom control was the most important attribute for caregivers with a child who has experienced vomiting as a side effect. These respondents strongly disliked a treatment by IV infusion every month
Child has no experience with vomiting (*n* = 97)	
Child has experience with vomiting (*n* = 110)	
*P* *value* = *0.037*	
**UK adolescents, *N* = 100**	
**Adolescent at median age or older**^**a**^	Those younger than median age placed significantly more importance on improvement in symptom control, whereas those at median age or older placed more importance on avoiding headaches than on the other attributes included in the study
Younger than median age (*n* = 26)	
At or older than median age (*n* = 74)	
*P* *value* = *0.005*	
	Additionally, the preference weight estimates indicate that adolescents younger than median age preferred an injection every week to tablets or syrup twice a week
**Adolescent identifies as female**	Adolescents who did not identify as female placed more importance on avoiding headaches, whereas adolescents who identified as female placed more importance on improvement in symptom control than on the other attributes included in the study. Additionally, adolescents who did not identify as female preferred tablets, syrup, or injections to IV
Does not identify as female (*n* = 80)	
Identifies as female (*n* = 20)	
*P* *value* = *0.080*	
**Adolescent has experience with methotrexate**	Preferences were not statistically systematically different across the 2 groups, probably due to small sample size; however, respondents with methotrexate experience considered avoiding headaches the most important attribute, while respondents without methotrexate experience considered improving symptom control the most important attribute
Has no experience with methotrexate (*n* = 58)	
Has experience with methotrexate (n = 42)	
*P* *value* = *0.206*	
**Adolescent has experience with biologics**	Although the sample size did not allow for identification of systematic differences in preferences, respondents without biologics experience valued efficacy and adverse event attributes about the same, while respondents who have experience with biologics placed the most value on avoiding headaches and improving symptom control. Respondents with biologics experience preferred tablets or syrup twice a day or an injection once a week to IV
Has no experience with biologics (*n* = 17)	
Has experience with biologics (*n* = 83)	
*P* *value* = *0.109*	
**Adolescent has experience with injections**	Adolescents who do not have experience with injections did not have strong preferences for any specific attribute included in the survey. In fact, there were no statistically significant differences between symptom control; time until next flare-up; stomachaches, nausea, and vomiting; and mode and frequency of administration. Additionally, adolescents who have experience with injections placed more relative importance on improvement in symptom control and avoiding headaches and less relative importance on mode and frequency of administration. Interestingly, adolescents with injection experience preferred injection every week to tablets or syrup twice a day
Has no experience with injections (*n* = 25)	
Has experience with injections (*n* = 75)	
*P* *value* = *0.002*	
**Adolescent has experience with headaches**	Adolescents who have no experience with headaches placed more relative importance on improvement in symptom control and avoiding headaches, whereas adolescents who have experienced headaches placed more relative importance on improvement in symptom control and mode and frequency of administration than on avoiding headaches
Has no experience with headaches (*n* = 47)	
Has experience with headaches (*n* = 53)	
*P* *value* = *0.007*	
**Adolescent has experience with stomachaches**	Adolescents who have no experience with stomachaches as a side effect of treatment placed more relative importance on improvement in symptom control and avoiding headaches, and adolescents who have experience with stomachaches as a side effect of their treatment d**id** not have strong preferences for any specific attribute included in the survey
Has no experience with stomachaches (*n* = 41)	
Has experience with stomachaches (*n* = 59)	
*P* *value* = *0.007*	
**Adolescent has experience with vomiting**	Adolescents who have no experience with vomiting placed more relative importance on improvements in symptom control and avoiding headaches than the other attributes included in the study. This group of adolescents did not have statistically different preferences across varying modes and frequencies of administration
Has no experience with vomiting (*n* = 62)	
Has experience with vomiting (*n* = 38)	
*P* *value* = *0.003*	Although it may appear that respondents with vomiting experience placed more relative value on mode and frequency of administration and symptom control than other attributes in the study, there were no statistical differences across any of the CRI estimates for any attribute. However, those with vomiting experience preferred treatment that is taken as tablets or syrup twice a day or injection every week over an IV infusion every month
**UK caregivers, *N* = 200**	
**Caregiver’s child’s median age**^**b**^	Those respondents with a child below the median age generally valued improvement in symptom control, avoiding stomachaches, and combination therapy
Child is younger than median age (*n* = 100)	
Child is at or older than median age (*n* = 100)	Those respondents with a child at or above the median age valued the improvement in symptom control the most relative to the other attributes in the study. They did not have statistically different preferences between no additional medicines and combination therapy, but this group preferred injections, tablets, or syrup to IV infusions
*P* *value* = *0.001*	
**Caregiver’s child’s gender**	Respondents with a child who did not identify as female generally placed the most importance on improvements in symptom control relative to the other attributes in the study. These respondents preferred a treatment that is taken by tablet, syrup, or injection over a treatment by IV infusion
Child does not identify as female (*n* = 131)	
Child identifies as female (*n* = 69)	
*P* *value* = *0.006*	Respondents with a child who identified as female generally placed more relative importance on improvements in symptom control, avoiding stomachaches, and having an additional medicine. These respondents preferred a combination therapy regimen over no additional medicines
**Caregiver’s level of education**	Caregivers with less than an undergraduate degree, on average, valued all of the attributes included in the study. These respondents preferred a regimen with additional medicines over a regimen that does not involve combination therapy, and they preferred tablets or syrup twice a day over an IV infusion every month and an injection every week (but no more than an injection every 2 weeks)
Caregiver has less than an undergraduate degree (*n* = 51)	
Caregiver has an undergraduate or higher degree (*n* = 149)	
	Those caregivers with an undergraduate degree or higher placed the most relative importance on improvements in symptom control. These respondents also preferred an additional medicine to no combination therapy, but they had no statistically greater preferences for tablets or syrup twice a day to another mode and frequency of administration
*P* *value* = *0.011*	
**Caregiver’s child has experience with methotrexate**	Caregivers with a child who had no experience with methotrexate placed most relative importance on improvements in symptom control; avoiding stomachaches, nausea, and vomiting; and having a combination therapy
Child has no experience with methotrexate (*n* = 108)	
	Caregivers with a child who does have experience with methotrexate generally placed the most relative importance on improvements in symptom control and avoiding headaches. Unlike those without methotrexate experience, they did not differentiate between no additional medicines or combination therapy
Child has experience with methotrexate (*n* = 92)	
*P* *value* = < *0.001*	
**Caregiver’s child has experience with biologics**	Caregivers with a child with no experience with biologics generally cared about improvements in symptom control and mode and frequency of administration. These respondents strongly preferred a treatment by tablet, syrup, or injection over a treatment by IV infusion, but they did not have statistically different preferences between a treatment with or without combination therapy
Child has no experience with biologics (*n* = 48)	
Child has experience with biologics (*n* = 152)	
*P* *value* = < *0.001*	
	Respondents with a child with biologics experience generally valued improvements in symptom control the most. These respondents did not prefer one mode of administration over another, but they did prefer a treatment with combination therapy
**Caregiver’s child has experience with injections**	Those respondents with a child with no experience with injections strongly valued improvements in symptom control over the other attributes included in the study. These respondents preferred tablets or syrup twice a day or an injection every 2 weeks over an IV infusion every month, but they did not prefer an injection every week over an IV infusion
Child has no experience with injections (*n* = 29)	
Child has experience with injections (*n* = 171)	
*P* *value* = *0.001*	
	Respondents with a child who does have experience with injections did not have preferences for any one attribute that dominated over preferences for other attributes. These respondents had a preference for an injection every week over an IV infusion every month and also preferred a treatment with combination therapy
**Caregiver’s child has experience with headaches**	Caregivers with a child without experience with headaches generally place more relative importance on improvements in symptom control and avoiding headaches than other attributes in the study
Child has no experience with headaches (*n* = 44)	
Child has experience with headaches (*n *= 156)	Caregivers with a child who does have experience with headaches also placed a great deal of relative importance on improvements in symptom control and avoiding headaches. Also, they preferred a treatment regimen that included additional medicines over one without additional medicines
*P* *value* = < *0.001*	
**Caregiver’s child has experience with stomachaches**	Respondents with a child who has not experienced stomachaches as a side effect placed greater relative importance on improvements in symptom control, time until next flare-up, and avoiding headaches. These respondents preferred a treatment by syrup or tablet over a treatment by infusion and preferred a treatment with no additional medicines to one that needs additional medicines
Child has no experience with stomachaches (*n* = 45)	
Child has experience with stomachaches (*n* = 155)	
	Respondents with a child who has experienced stomachaches as a side effect similarly valued improvements in symptom control, but they also valued avoiding stomachaches, nausea, and vomiting. These respondents also preferred a treatment by syrup, tablet, or injection over a treatment by infusion, but they preferred a treatment with additional medicines to one without additional medicines
*P* *value* = < *0.001*	
**Caregiver’s child has experience with vomiting**	Respondents with a child who has not experienced vomiting as a side effect strongly preferred improvements in symptom control relative to the other variables included in the study
Child has no experience with vomiting (*n* = 69)	
Child has experience with vomiting (*n* = 131)	
	Respondents with a child who had experience with vomiting did not, on average, have any preferences that dominated other attributes in terms of conditional relative importance. Unlike those with no vomiting experience, these respondents preferred a treatment regimen with additional medicines over one that does not include additional medicines
*P* *value* = < *0.001*	

*CRI* Conditional relative importance, *IV* Intravenous, *JIA* Juvenile idiopathic arthritis

^a^Respondents were asked in the survey to provide their age. The median age was identified at 15 years old

^b^Respondents were asked in the survey to provide the age of their child with JIA. The median age was identified at 13 years old

For the UK cohorts, results of the subgroup analyses revealed a significant amount of preference heterogeneity across multiple sample characteristics for both adolescents and caregivers (Table 3 and Figures S18-S34, and Supplemental Appendix B). For the adolescent samples, systematically different preferences were found across subgroups defined by age, injection experience, headache experience, stomachache experience, and vomiting experience. For the caregiver samples, systematically different preferences were found across the subgroups defined by the age of the child, the gender of the child, the caregiver’s educational attainment, the child’s experience with methotrexate, the child’s experience with biologics, the child’s experience with headaches, the child’s experience with stomachaches, the child’s experience with vomiting, and the child’s experience with injections.

## Discussion

This DCE survey assessed how adolescent patients with JIA and caregivers of adolescent and child patients with JIA in the US and UK prioritise treatment benefits (improvements in symptom control and increasing the time until the next flare-up) relative to treatment characteristics, such as mode and frequency of administration, the need for combination therapy, and treatment-related adverse events (AEs) (stomachache, nausea, and vomiting; headache). Across all samples, improvement in symptom control was as important as or more important than the other study attributes. In the US, preferences for other attributes were generally similar across caregivers and adolescents; however, adolescents were more focused on avoiding AEs (particularly stomachache) while caregivers were more focused on onset of action. This can perhaps be explained by the differences between the adolescent and the older adult brain, with the adolescent brain preferring to focus on the present rather than potential future consequences [25]. Preferences in the UK were less consistent across caregivers and adolescents. Adolescents placed the same importance on symptom control and avoiding headache, while for caregivers symptom control was the most important attribute and the other attributes had similar levels of importance. This could be an effect of sample size, as there were only 100 UK adolescent respondents. Further, UK adolescents appeared to prefer combination therapy to no combination therapy; however, the difference between these 2 options was not statistically significant. UK caregivers also preferred treatments that require combination therapy over those that do not require combination therapy. This somewhat counterintuitive finding could possibly be explained by previous experience with combination therapy, or because the respondents interpreted use of additional medicine as an indicator of greater efficacy.

The results from this study offer insight into the treatment preferences of adolescents with JIA and caregivers of children and adolescents with JIA, which previous research has shown to be an important factor in pediatric rheumatologists’ treatment decisions [26]. Overall, adolescents and caregivers value improvements in symptom control and decreasing the time until the next flare-up. The results also speak to some potential cultural differences (e.g., clinical practice patterns, access to medicines, payer systems) between the US and UK samples with respect to preferences for some attributes. In particular, caregivers in the UK preferred a treatment with additional medicines, as opposed to US caregivers, who were indifferent between treatments with and without additional medicines.

There were also regional differences between the adolescent samples in the relative importance of the attributes of stomachache (to which US adolescents were more averse than UK adolescents) and headache (to which UK adolescents were far more averse than US adolescents, considering this the most important attribute relative to the others evaluated). Importantly, US adolescents and caregivers were indifferent to mode of administration, whereas UK adolescents and caregivers were both averse to IV infusions. Biologic infusions are often administered in a hospital setting, typically only during weekdays, so children and adolescents with JIA may need to travel some distance to the hospital and miss at least half a day of school. These factors may influence the aversion to IV infusions. Broadly, preferences regarding mode of administration suggest that patients and caregivers in the US and UK are amenable to injections for a treatment that will improve symptoms. Patient and caregiver preferences may reflect samples of treatment-experienced respondents who have previously received an injectable treatment and therefore may not have the same fears as injection-naïve patients may have.

Aside from geographic variation, the findings also indicate significant heterogeneity in preferences associated with a range of observable characteristics (e.g., treatment and symptom experience). Specifically, among US adolescents, varying preferences were found for subgroups defined by gender, methotrexate experience, headache experience, and stomachache experience, whereas for US caregivers, varying preferences were found only across the subgroup defined by the caregiver’s child’s experience with vomiting. Among UK respondents, varying preferences were found across adolescent subgroups defined by age, injection experience, headache experience, stomachache experience, and vomiting experience. For UK caregivers, varying preferences were found across the subgroups defined by the age or gender of the child; the caregiver’s educational attainment; and the child’s experience with methotrexate, biologics, injections, headaches, stomachaches, and vomiting. These results emphasise that sociodemographic and clinical characteristics and experiences with treatment may influence patient and caregiver preferences and should be considered in discussions of individualised treatment plans with families.

The DCE has a number of strengths derived from the use of best practices [13]. In particular, the survey was carefully designed and pretested using in-depth interviews with participants and used an experimental design developed using good research practices [11]. The treatment-choice data were analysed using advanced RPL methods following good research practices [12, 27] that avoid estimation bias from both unobserved variation in preferences across the sample and within-sample correlation in the choice sequence for each respondent. Our findings also support the idea that preferences for paediatric diseases can be successfully captured from adolescents and caregivers, and thus provide insights into potential areas of discordance between preferences for these populations.

Nonetheless, the study has limitations. The study used purposive sampling to recruit adolescent and caregiver samples for whom the hypothetical premise of the DCE would be most salient; thus, the respondents’ preferences may not be representative of those affected by JIA in the US and UK. In addition, while the sample size targets for this study were deemed sufficient to produce precise results and were consistent with recommendations from the literature, the final samples, especially those in the UK, were more heterogeneous than expected and may not be completely representative of the JIA population. Sample sizes for some subgroups were small, potentially limiting the ability to detect statistically significant differences in preferences. The adolescent samples in the US and particularly in the UK were predominantly male, which is not consistent with the broader JIA population. Further, the results are subject to potential volunteer bias and to potential information bias, and the perspectives of individuals without access to technology are not reflected. Additional factors, such as socioeconomic status and access to care, were not collected in our study and may influence treatment preferences among the broader population of individuals affected by JIA. Because all data, including JIA diagnosis and clinical characteristics, were self-reported and were not clinically validated, there is the potential for misclassification of some patients. Respondents were making hypothetical treatment decisions, which may not predict actual decisions made in a clinical setting. Finally, the survey was conducted during the coronavirus disease 2019 pandemic, and respondents may have responded to the survey differently during this period than during other times.

## Conclusions

For adolescents with JIA and caregivers of children and adolescents with JIA in the US and UK, a treatment that considerably improves symptom control, prolongs the time until the next flare-up, and avoids AEs such as headaches, stomachaches, nausea, and vomiting is desirable. On average, adolescents and caregivers are less concerned with difference in modes and frequencies of administration. Results of this study, which demonstrate adolescent patients’ and caregivers’ priorities for JIA treatment, could be used to inform a patient-centric care framework in JIA.

### Supplementary Information

**Additional file 1. **

## Data Availability

The data underlying this article will be shared on reasonable request to Joseph C. Cappelleri at joseph.c.cappelleri@pfizer.com.

## Abbreviations

AE: Adverse event
CRI: Conditional relative importance
csDMARD: Conventional synthetic disease-modifying antirheumatic drug
DCE: Discrete-choice experiment
IV: Intravenous
JAK: Janus kinase
JIA: Juvenile idiopathic arthritis
RPL: Random-parameters logit
tsDMARD: Targeted synthetic disease-modifying antirheumatic drug
UK: United Kingdom
US: United States
