# Supplementary material for: Adolescent and caregiver preferences for juvenile idiopathic arthritis treatment: a discrete-choice experiment
Source: Pediatr Rheumatol Online J. 2023 Oct 21;21:129. doi: 10.1186/s12969-023-00906-8 (PMC10589988; doi:10.1186/s12969-023-00906-8)
Supplement: Supplementary file 1 — Additional file 1. [file 12969_2023_906_MOESM1_ESM.pdf]

SUPPLEMENTAL APPENDIX

Appendix A: Subgroup Analyses, US Sample

Figure S1. Preference Weights and Conditional Relative Importance Estimates: Adolescents Younger Than Median Age Versus Adolescents at or Older Than Median Age

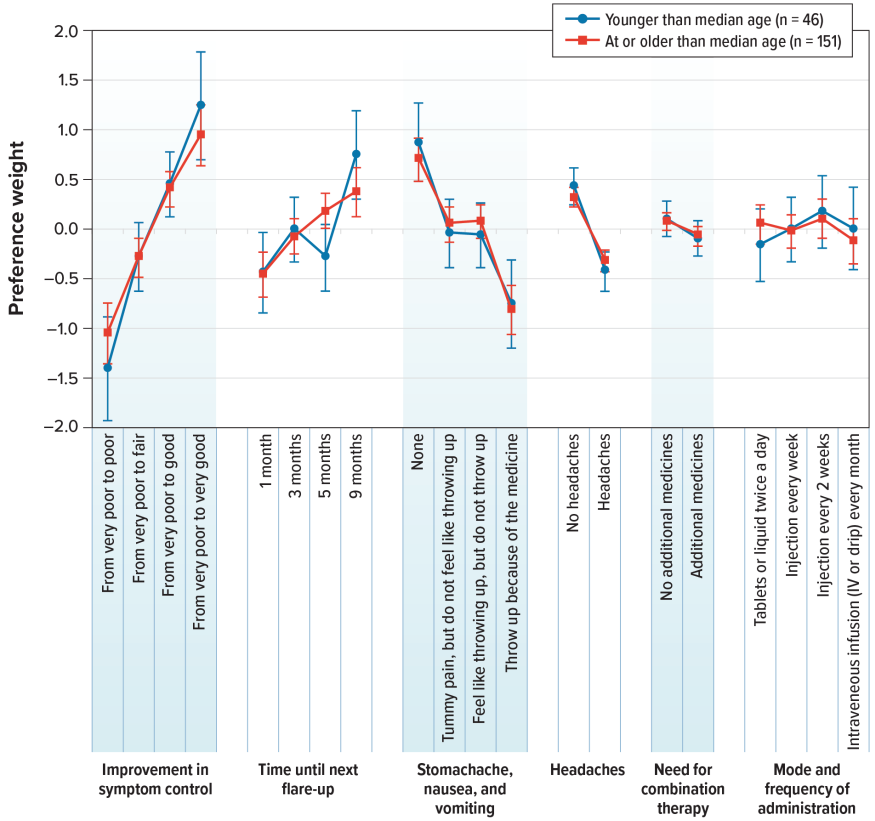

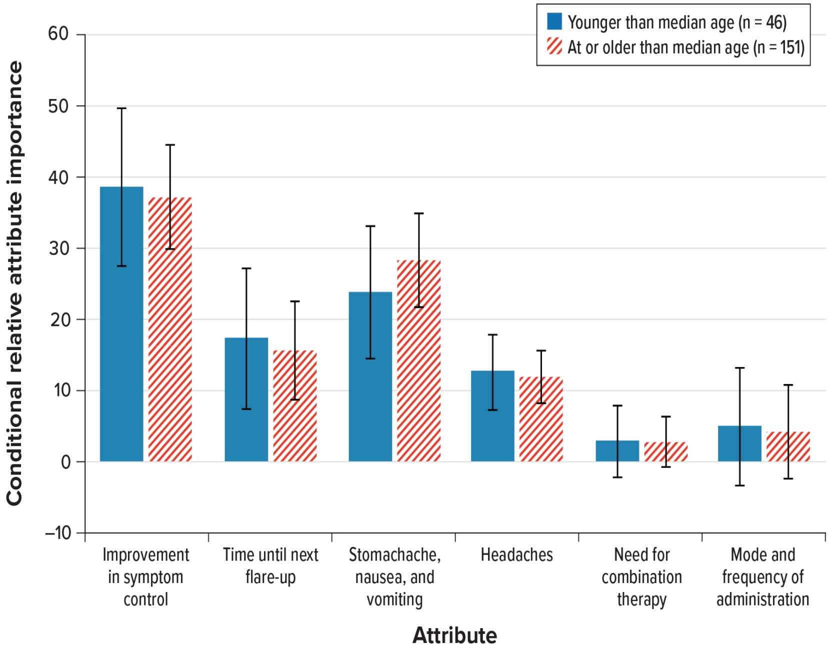

IV = intravenous.

Figure S2. Preference Weights and Conditional Relative Importance Estimates: Adolescents Who Identify as Female Versus Adolescents Who Do Not Identify as Female

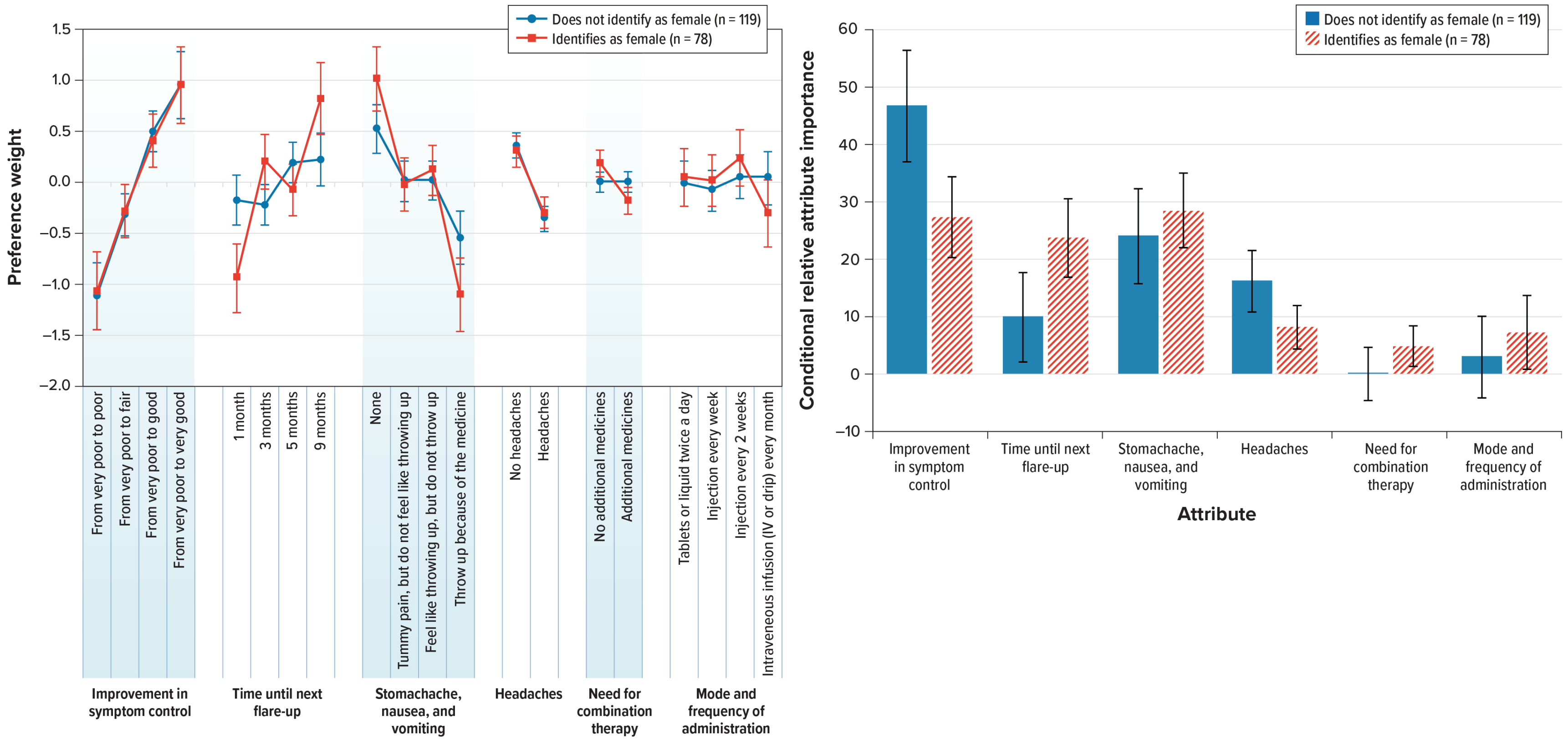

IV = intravenous.

Figure S3. Preference Weights and Conditional Relative Importance Estimates: Adolescents With Methotrexate Experience Versus Adolescents With No Experience With Methotrexate

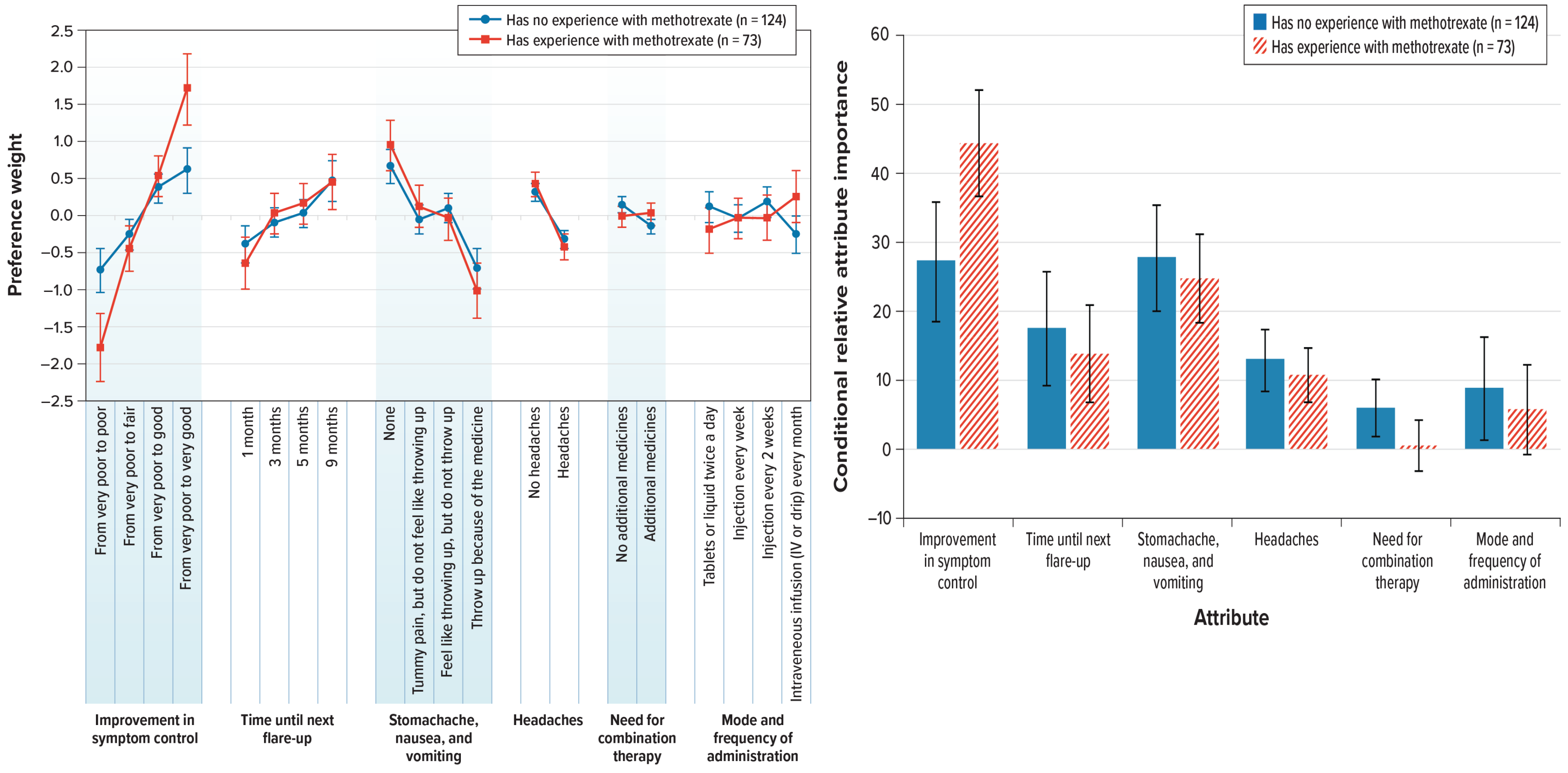

IV = intravenous.

Figure S4. Preference Weights and Conditional Relative Importance Estimates: Adolescents With Biologics Experience Versus Adolescents With No Experience With Biologics

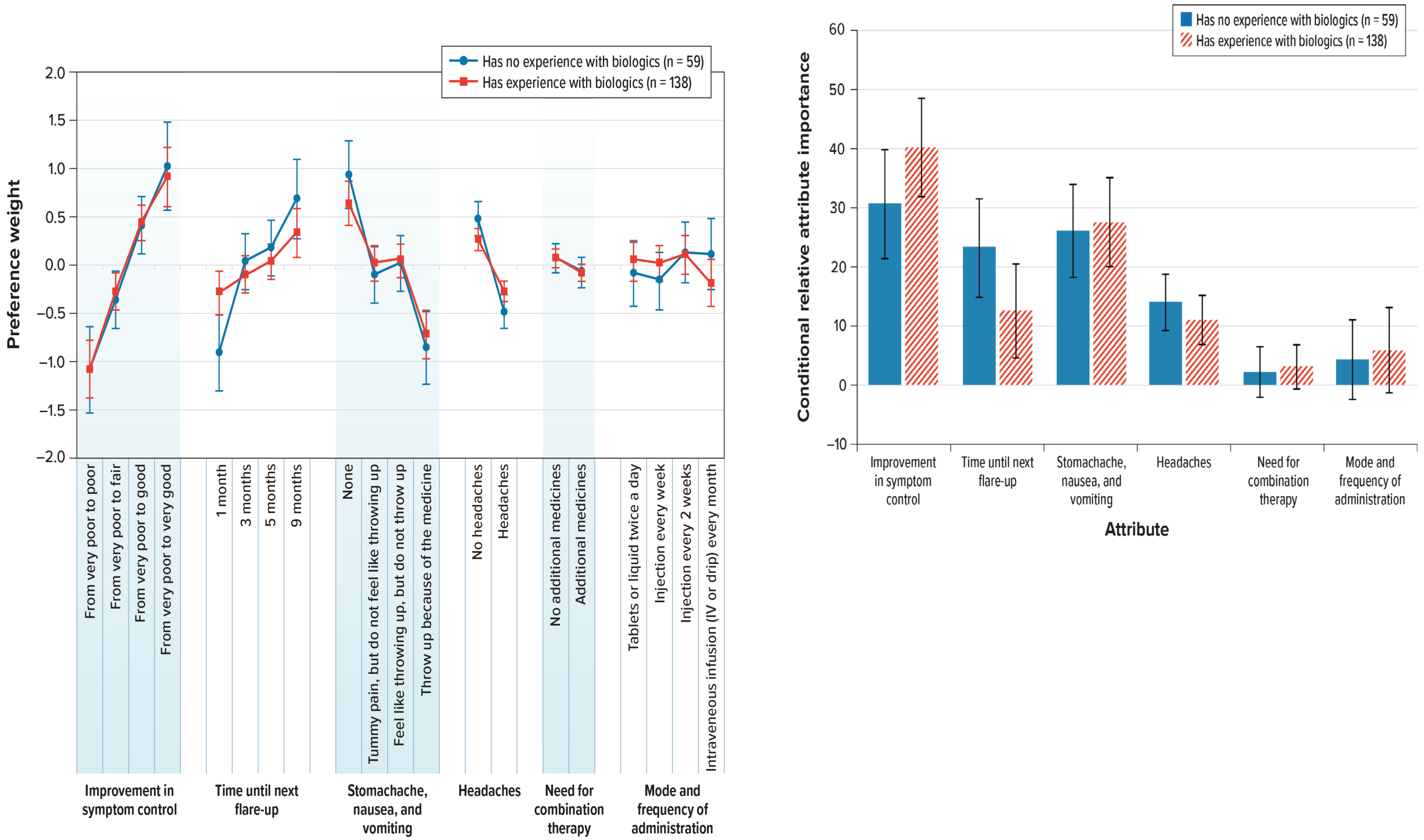

IV = intravenous.

Figure S5. Preference Weights and Conditional Relative Importance Estimates: Adolescents With Injection Experience Versus Adolescents With No Injection Experience

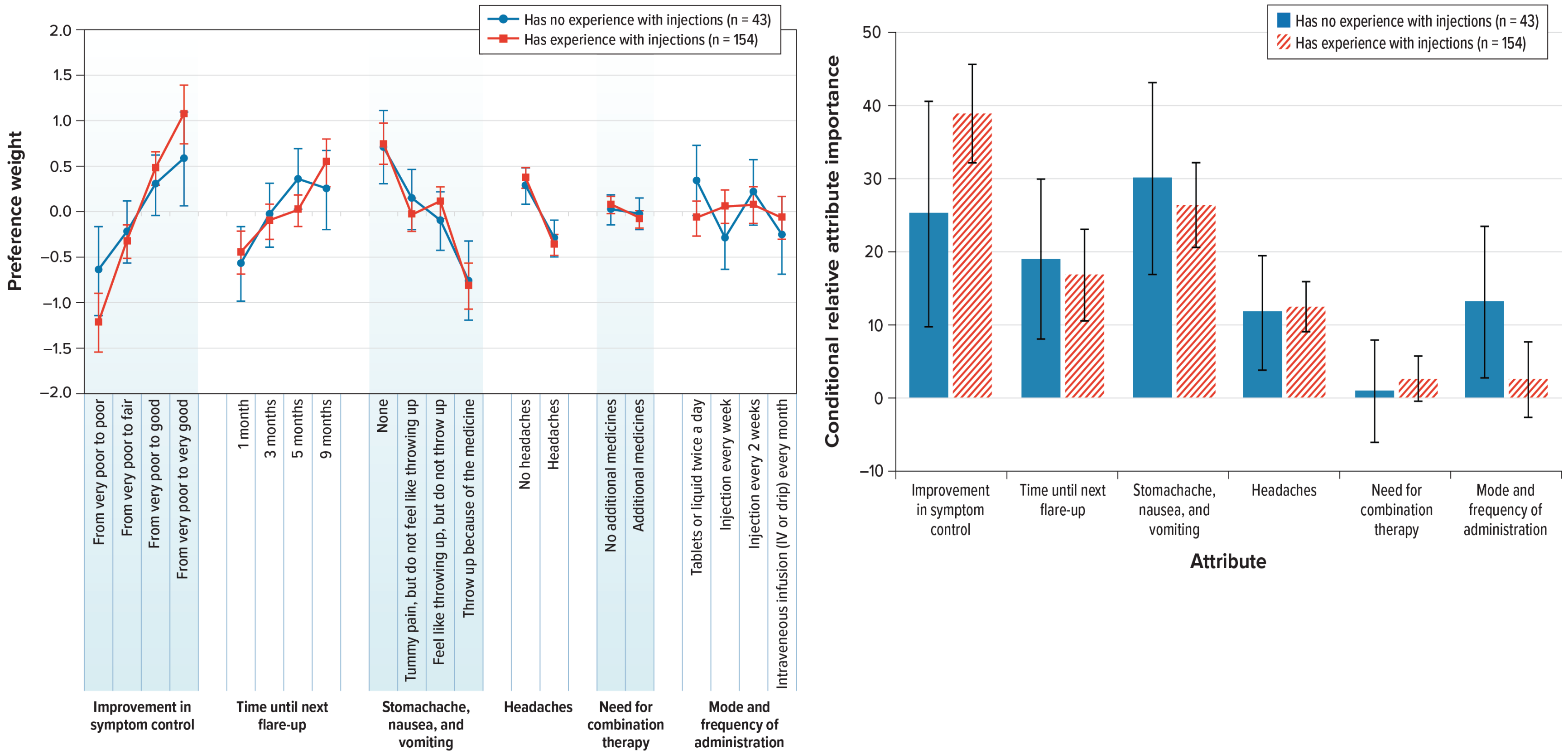

IV = intravenous.

Figure S6. Preference Weights and Conditional Relative Importance Estimates: Adolescents Who Have Experienced Headaches as a Side Effect of Their Treatment Versus Adolescents With No Experience With Headaches as a Side Effect of Their Treatment

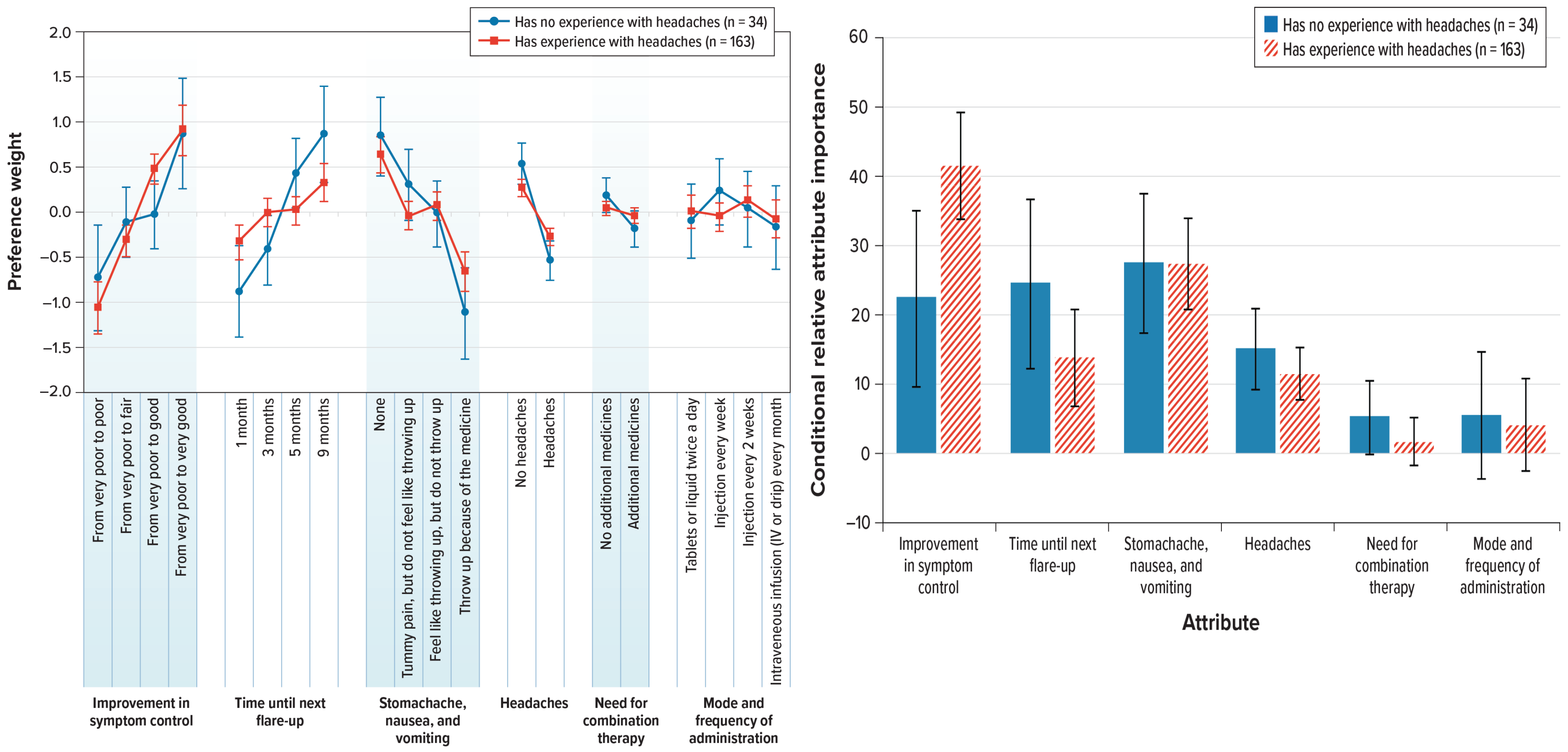

IV = intravenous.

Figure S7. **Preference Weights and Conditional Relative Importance Estimates: Adolescents Who Have Experienced Stomachaches as a Side Effect of Their Treatment Versus Adolescents With No Experience With Stomachaches as a Side Effect of Their Treatment**

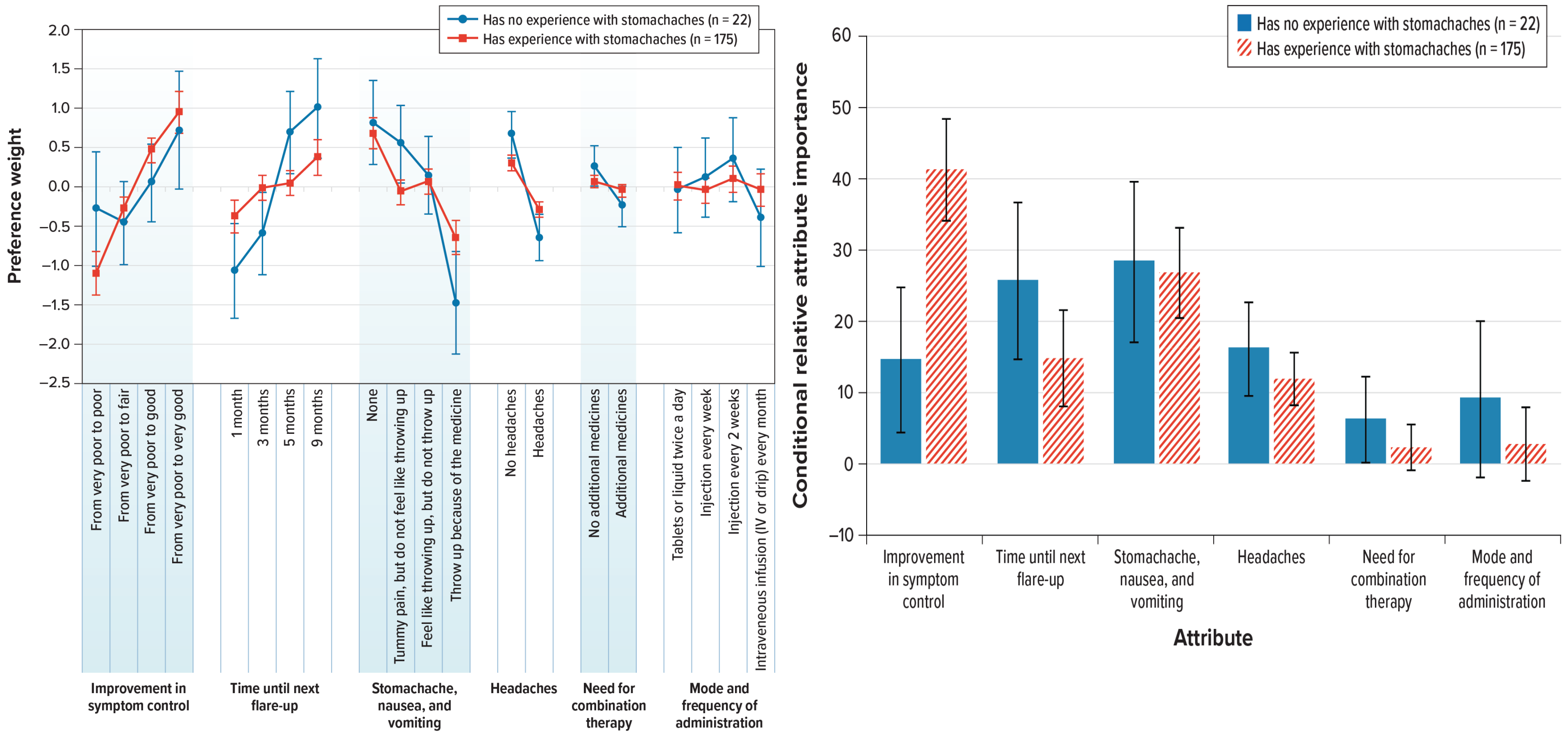

IV = intravenous.

Figure S8. Preference Weights and Conditional Relative Importance Estimates: Adolescents Who Have Experienced Vomiting as a Side Effect of Their Treatment Versus Adolescents With No Experience With Vomiting as a Side Effect of Their Treatment

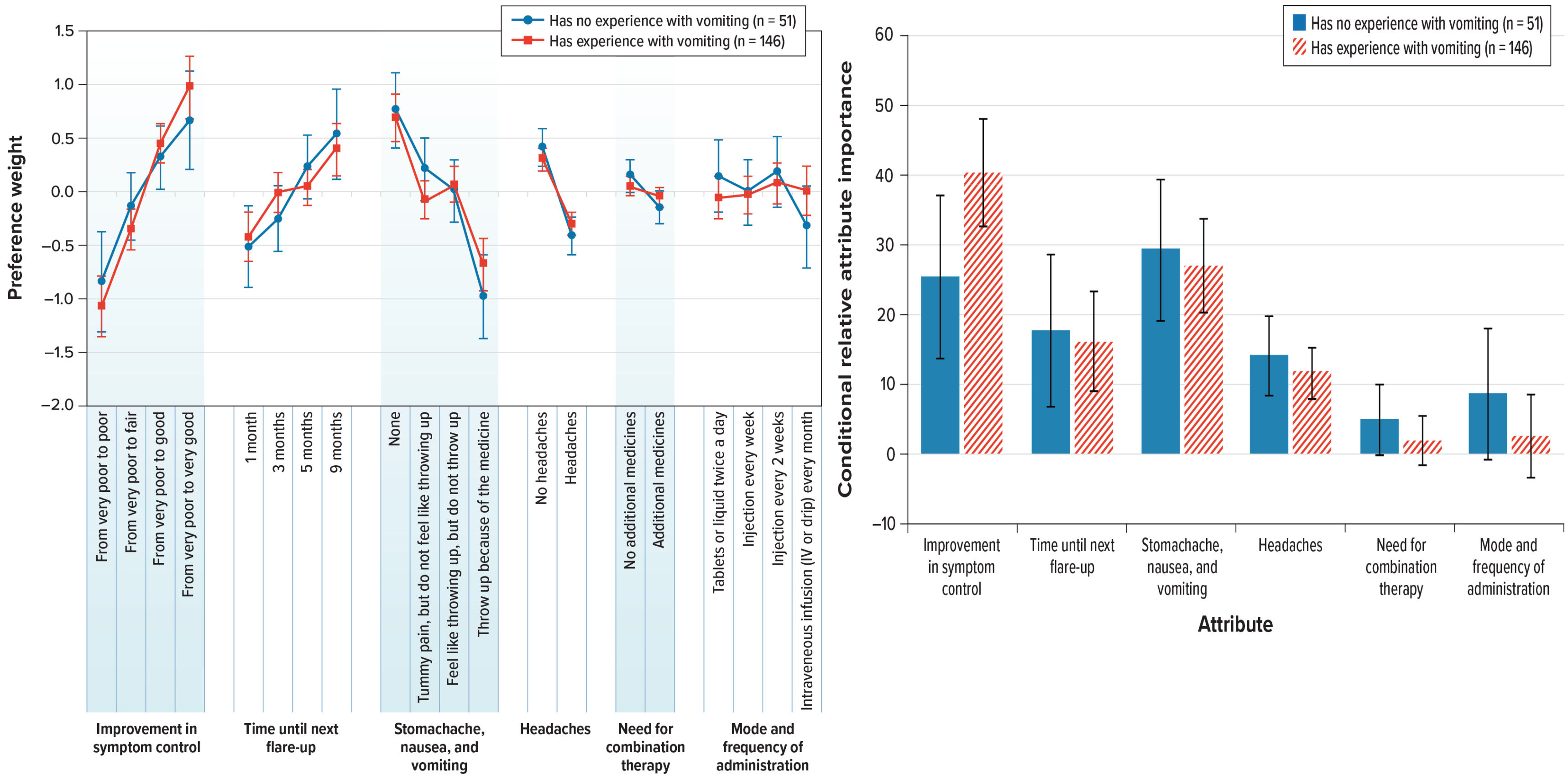

IV = intravenous.

Figure S9 Preference Weights and Conditional Relative Importance Estimates: Caregivers With a Child Younger Than Median Age Versus Caregivers With a Child at or Older Than Median Age

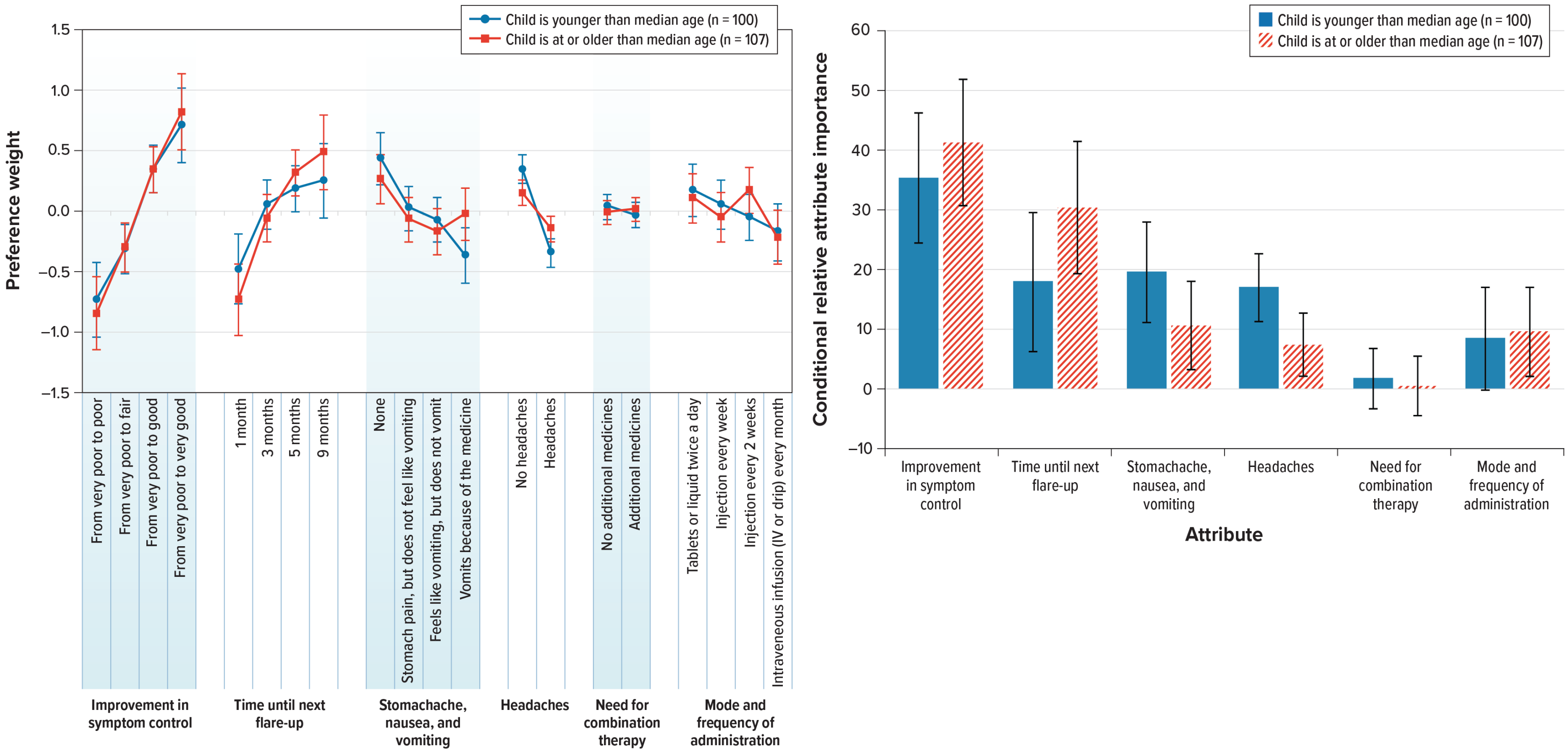

IV = intravenous.

Figure S10. Preference Weights and Conditional Relative Importance Estimates: Caregivers With a Child Who Identifies as Female Versus Caregivers With a Child Who Does Not Identify as Female

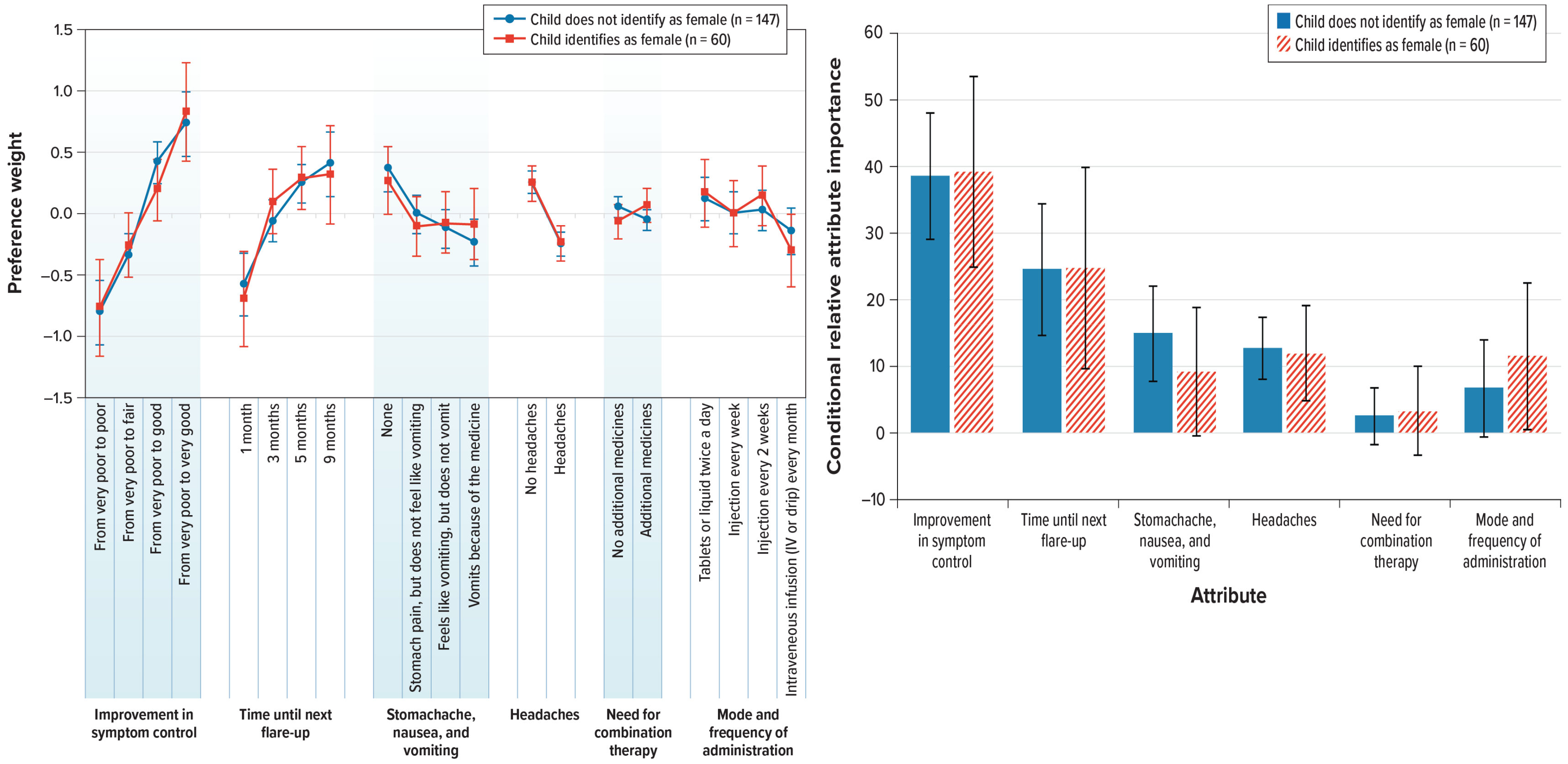

IV = intravenous.

Figure S11. Preference Weights and Conditional Relative Importance Estimates: Caregivers With a 4-Year Degree or Higher Versus Caregivers With Less Than a 4-Year Degree

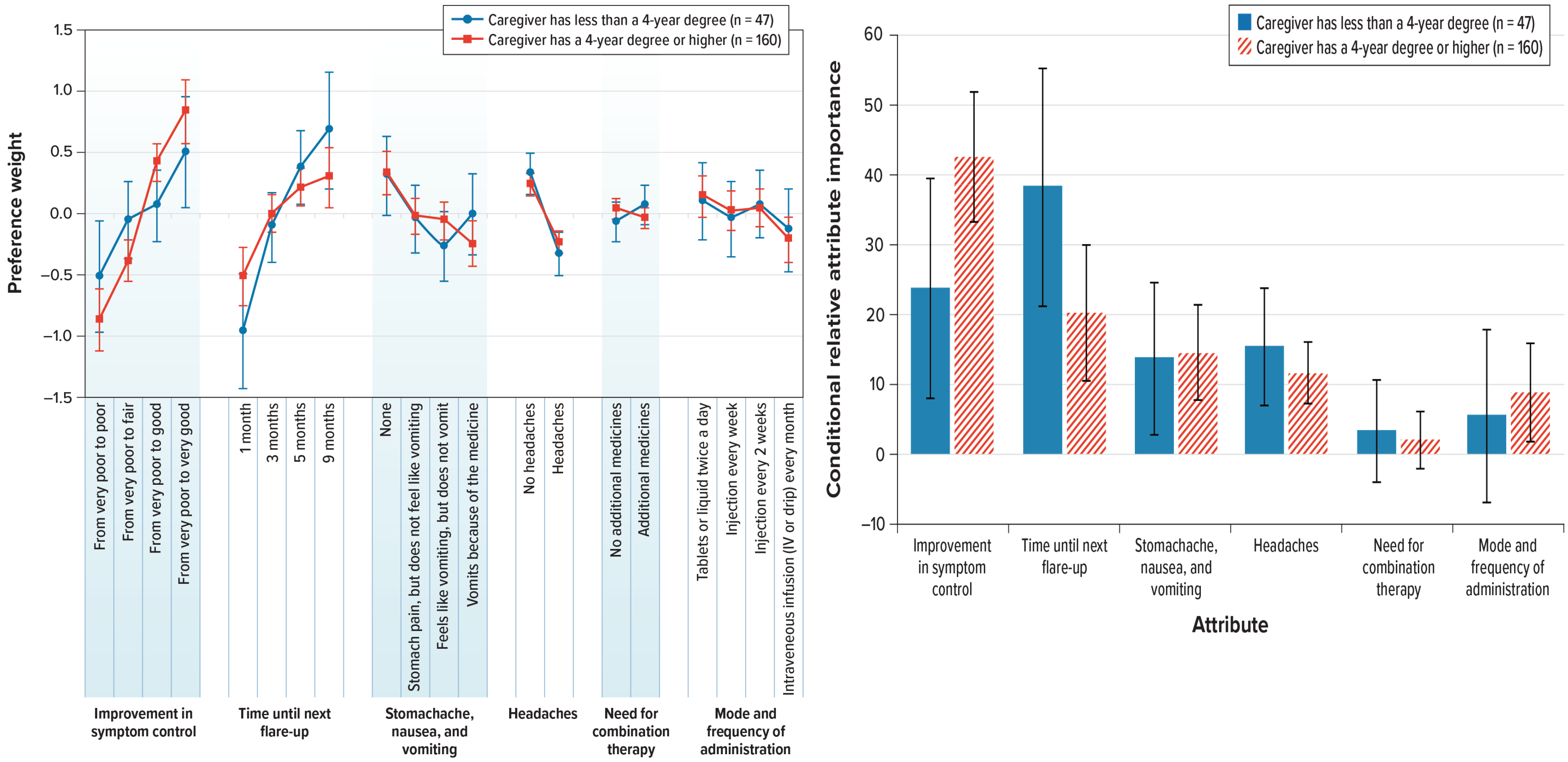

IV = intravenous.

Figure S12. Preference Weights and Conditional Relative Importance Estimates: Caregivers With a Child Who Has Experience With Methotrexate Versus Caregivers With a Child Who Has No Experience With Methotrexate

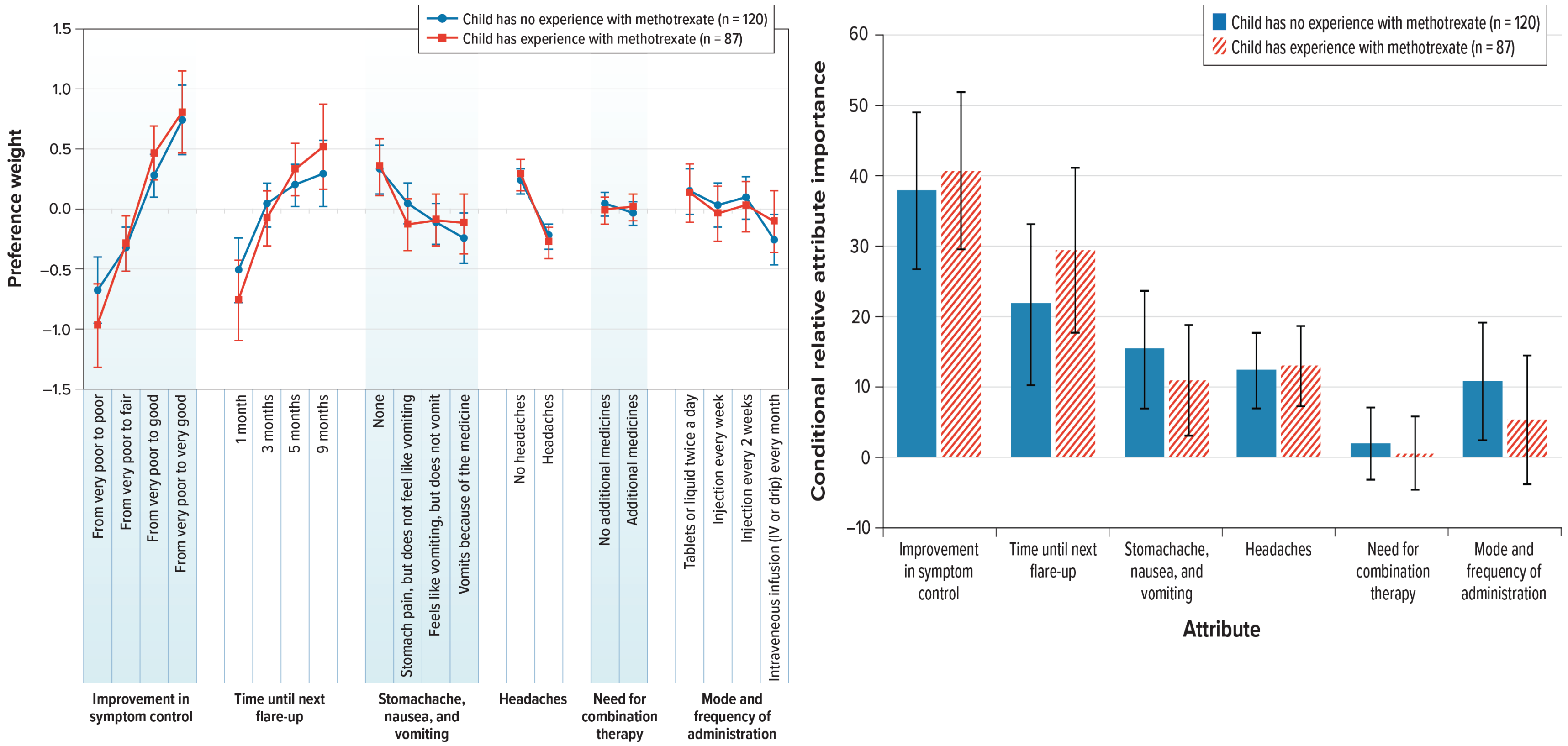

IV = intravenous.

Figure S13. Preference Weights and Conditional Relative Importance Estimates: Caregivers With a Child Who Has Experience With Biologics Versus Caregivers With a Child Who Has No Experience With Biologics

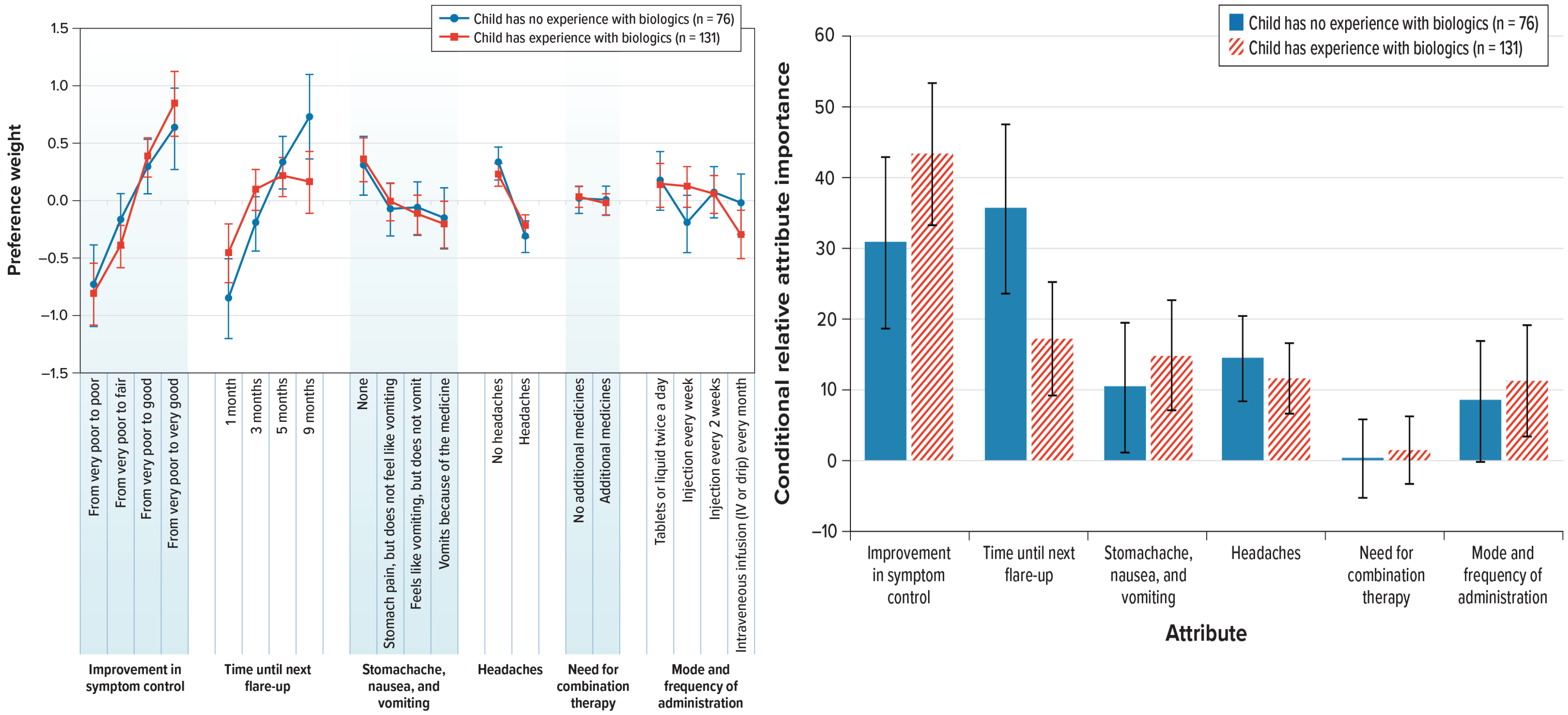

IV = intravenous.

Figure S14. Preference Weights and Conditional Relative Importance Estimates: Caregivers With a Child Who Has Experience With Injections Versus Caregivers With a Child Who Has No Experience With Injections

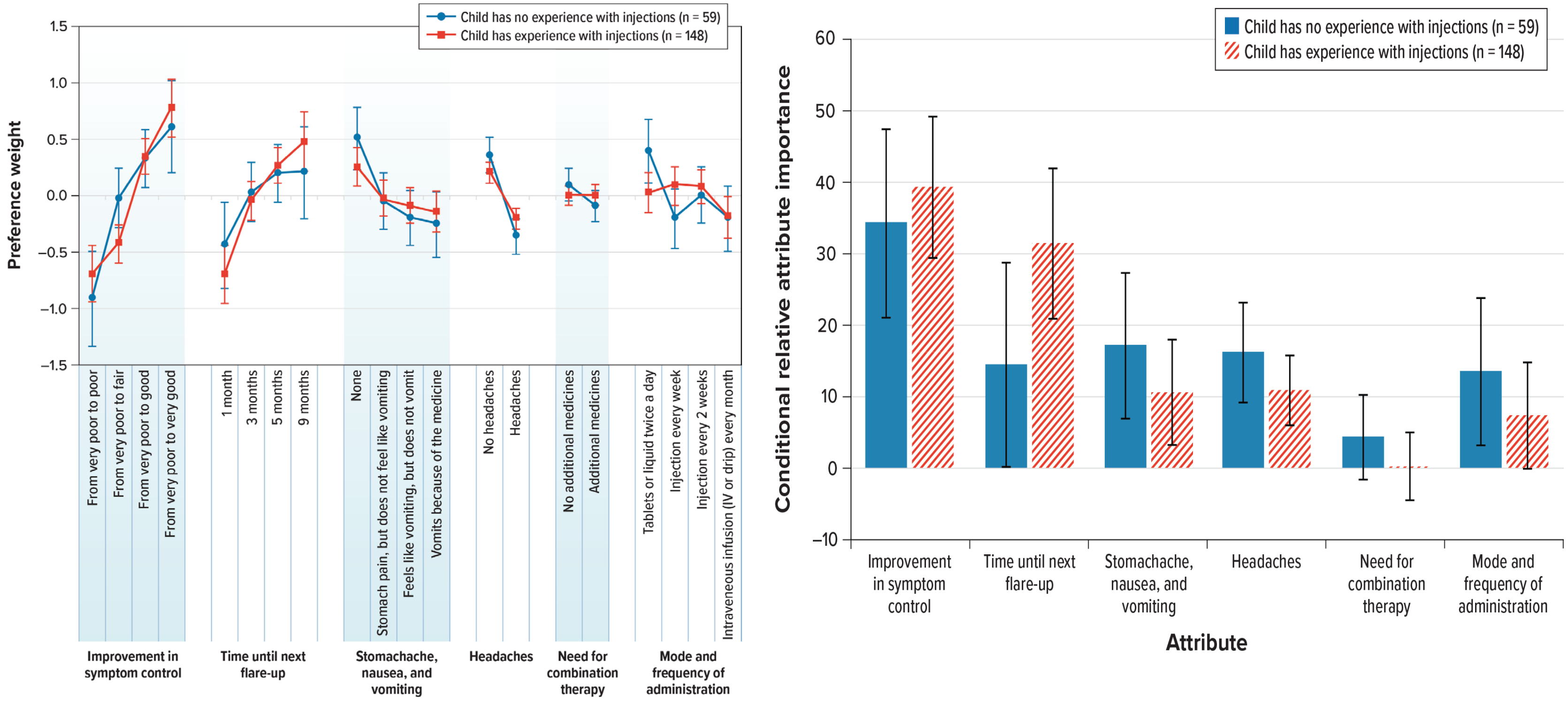

IV = intravenous.

Figure S15. Preference Weights and Conditional Relative Importance Estimates: Caregivers With a Child Who Has Experience With Headaches as a Side Effect of Their Treatment Versus Caregivers With a Child Who Has No Experience With Headaches as a Side Effect of Their Treatment

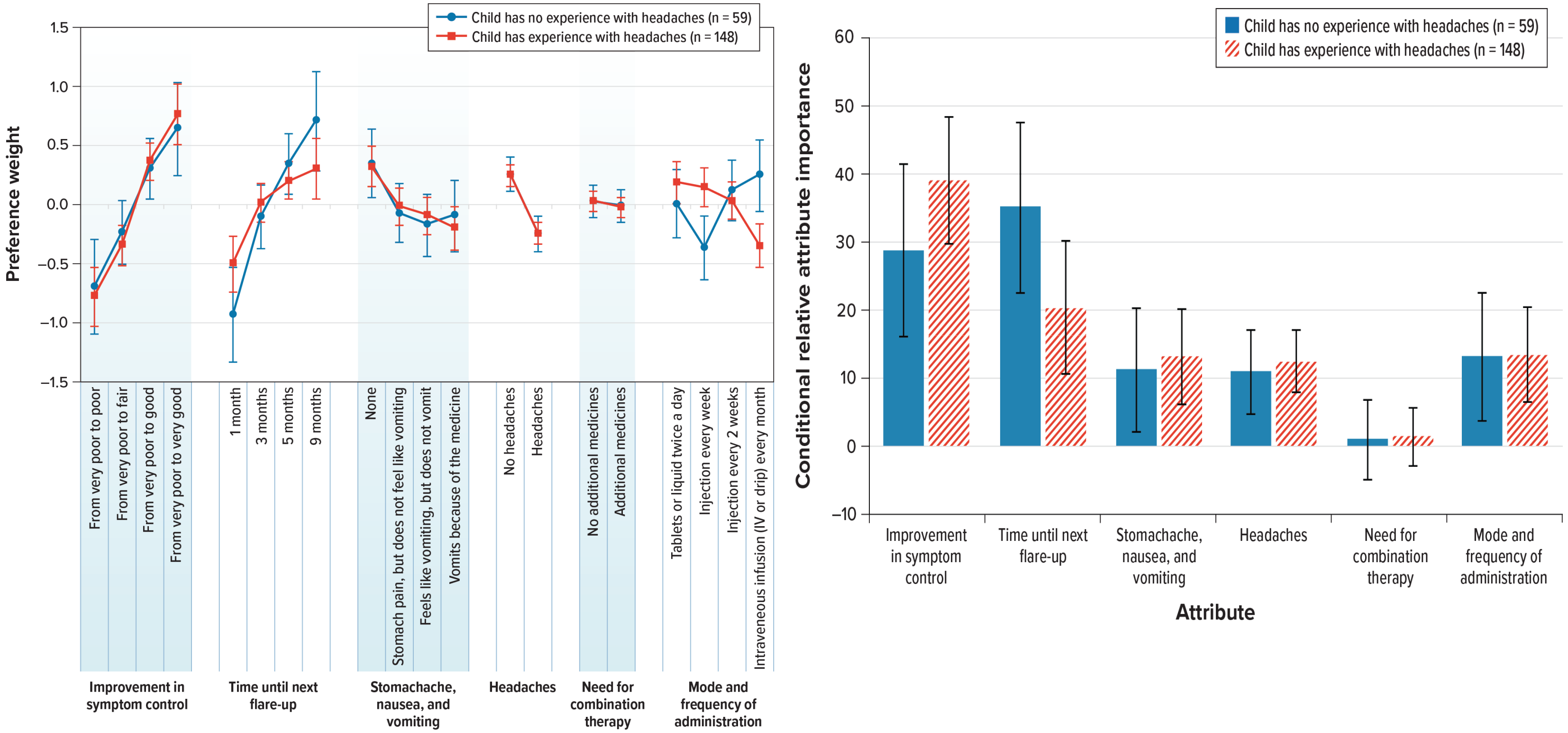

IV = intravenous.

Figure S16. Preference Weights and Conditional Relative Importance Estimates: Caregivers With a Child Who Has Experience With Stomachaches as a Side Effect of Their Treatment Versus Caregivers With a Child Who Has No Experience With Stomachaches as a Side Effect of Their Treatment

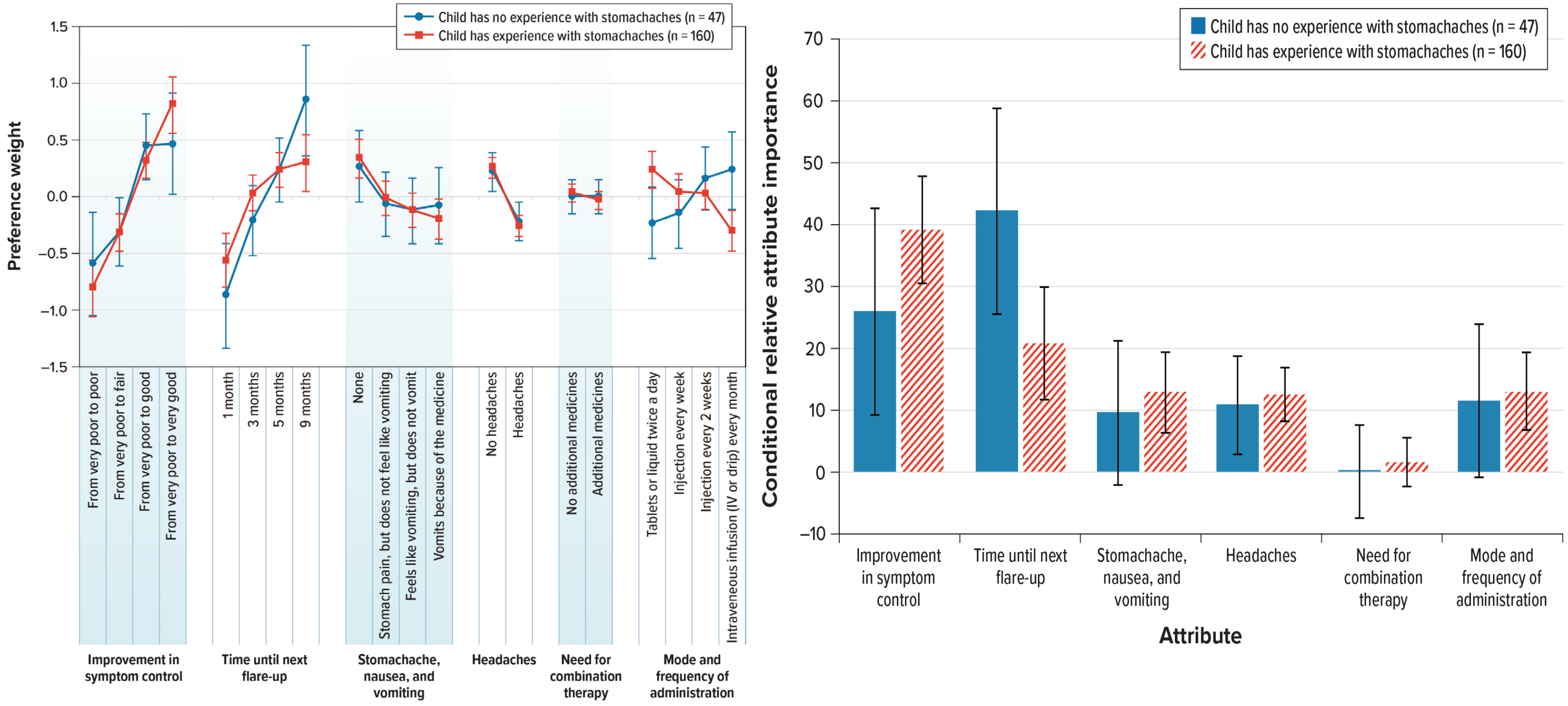

IV = intravenous.

Figure S17. Preference Weights and Conditional Relative Importance Estimates: Caregivers With a Child Who Has Experience With Vomiting as a Side Effect of Their Treatment Versus Caregivers With a Child Who Has No Experience With Vomiting as a Side Effect of Their Treatment

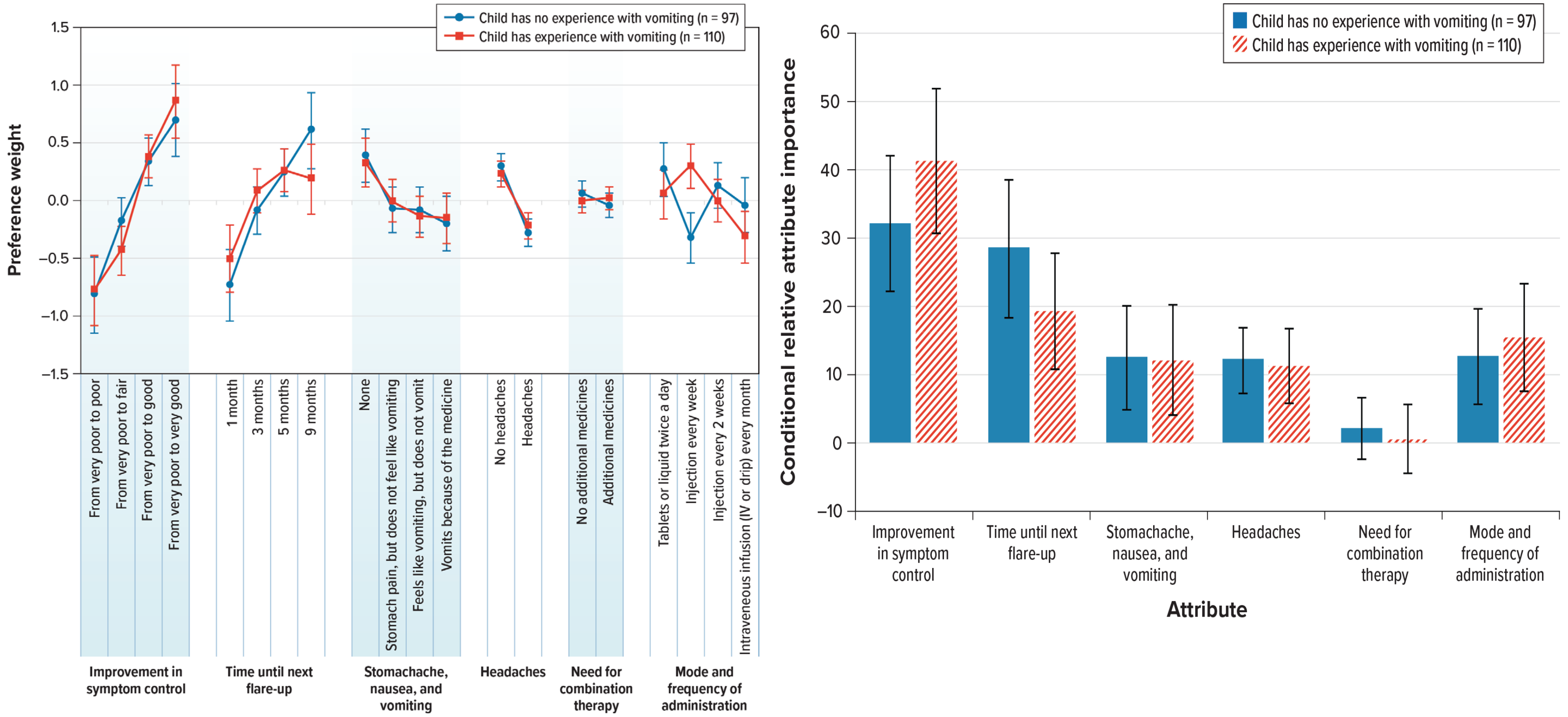

IV = intravenous.

Appendix B: Subgroup Analyses, UK Sample

Figure S18. Preference Weights and Conditional Relative Importance Estimates: Adolescents Younger Than Median Age Versus Adolescents at or Older Than Median Age

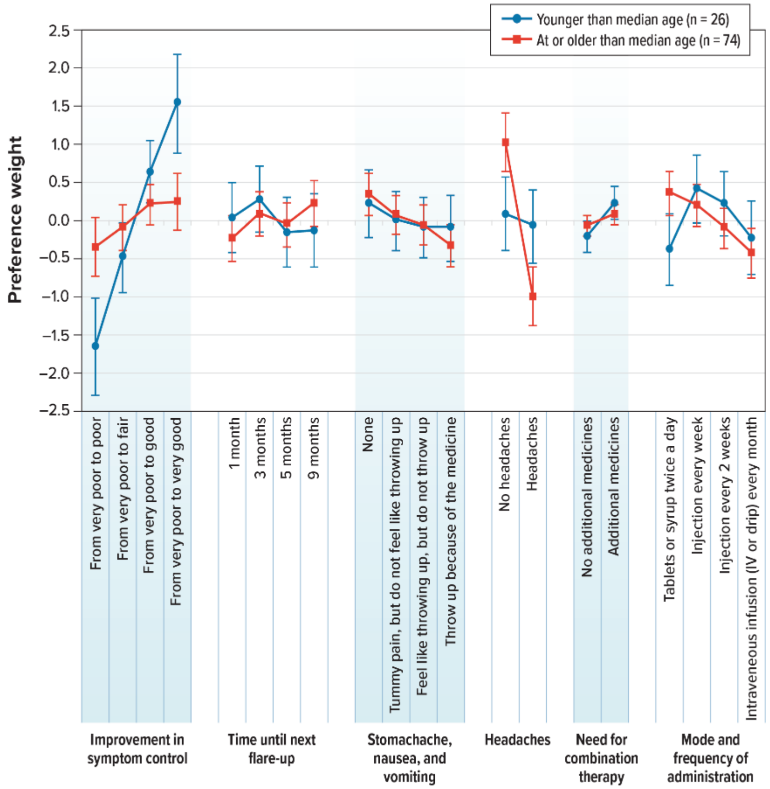

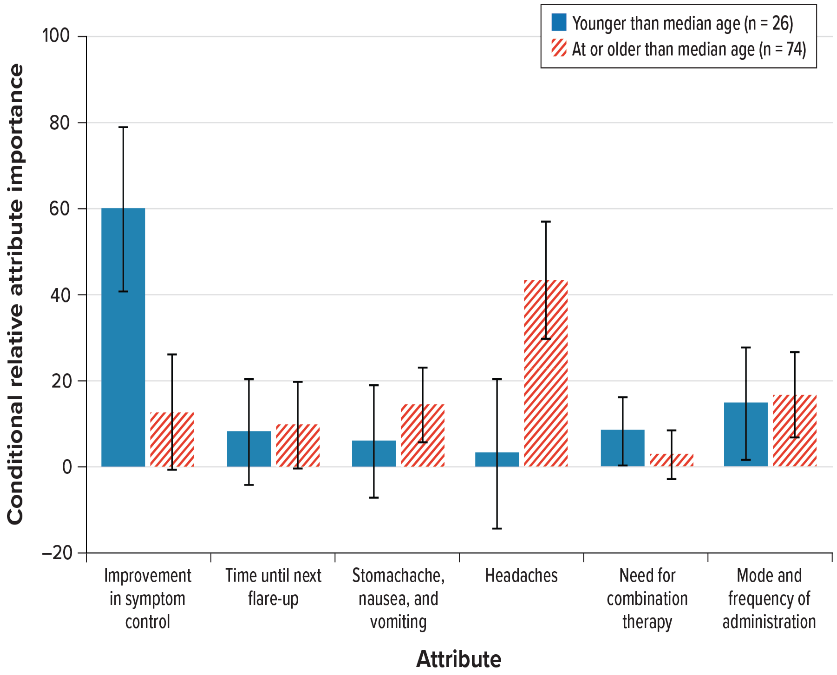

IV = intravenous.

Figure S19. Preference Weights and Conditional Relative Importance Estimates: Adolescents Who Identify as Female Versus Adolescents Who Do Not Identify as Female

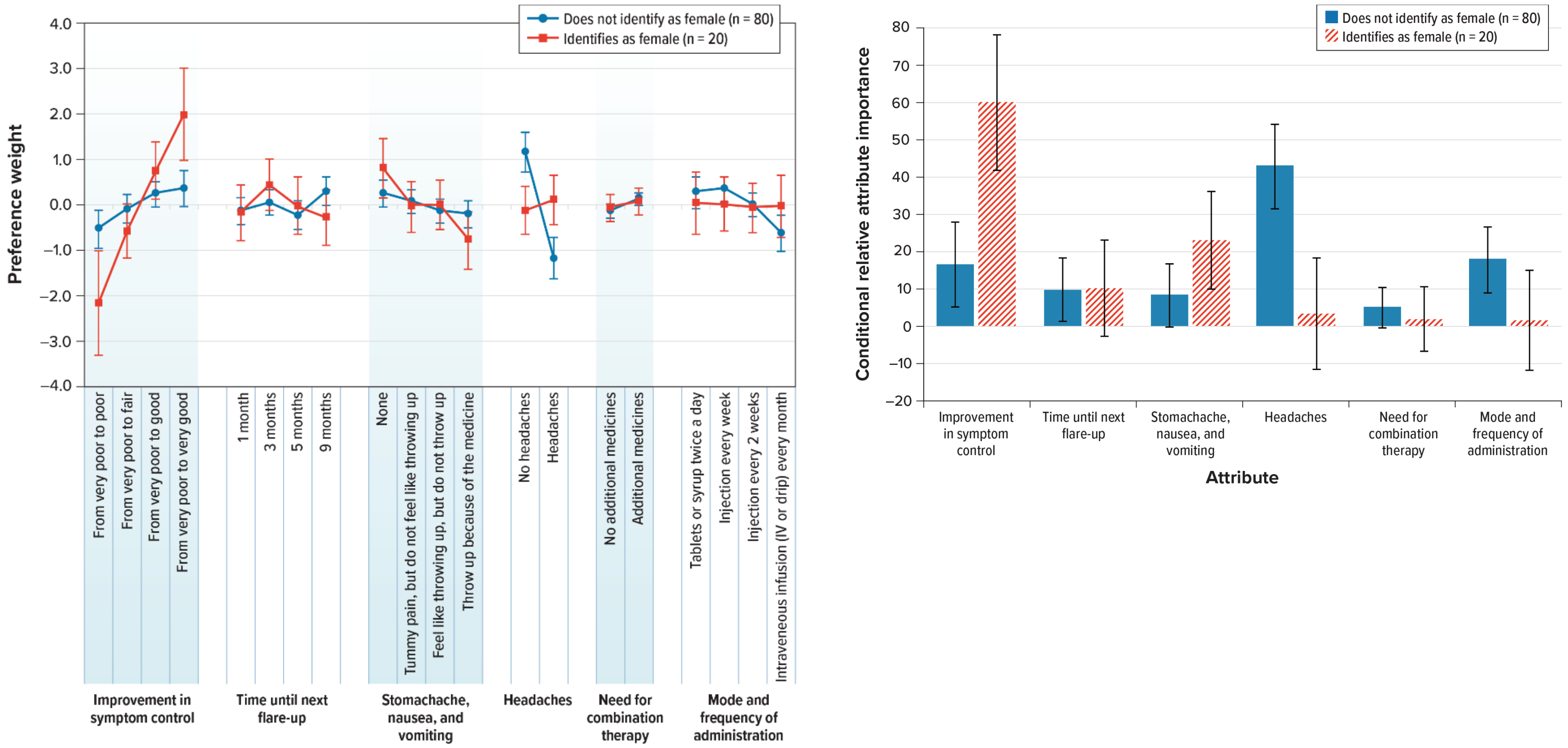

IV = intravenous.

Figure S20. Preference Weights and Conditional Relative Importance Estimates: Adolescents With Methotrexate Experience Versus Adolescents With No Experience With Methotrexate

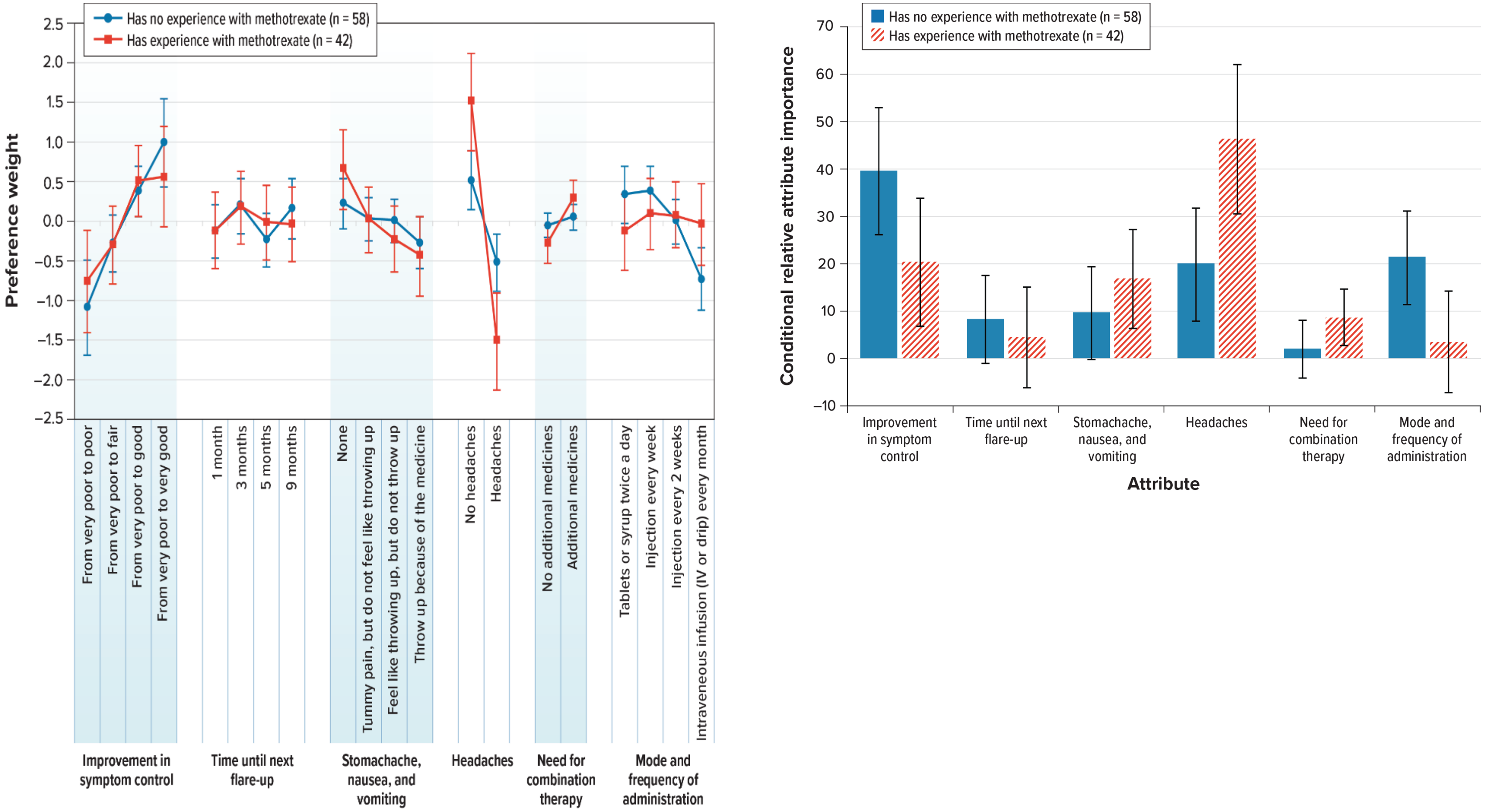

IV = intravenous.

Figure S21. Preference Weights and Conditional Relative Importance Estimates: Adolescents With Biologics Experience Versus Adolescents With No Experience With Biologics

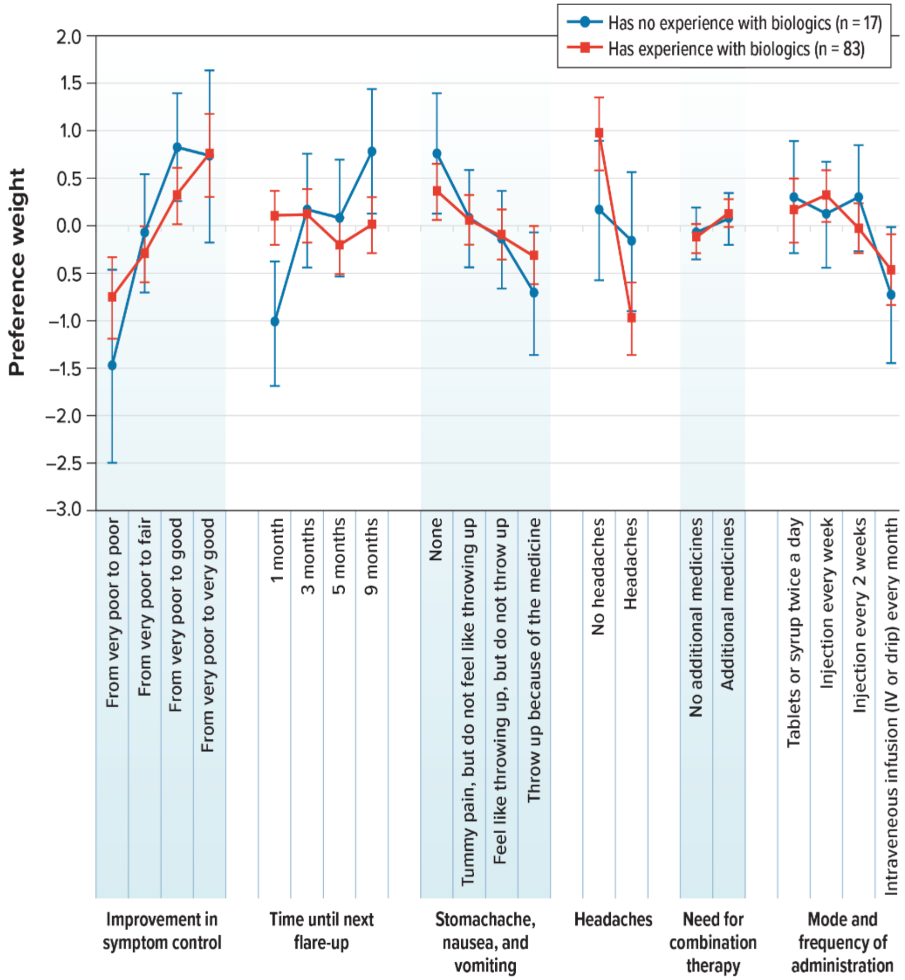

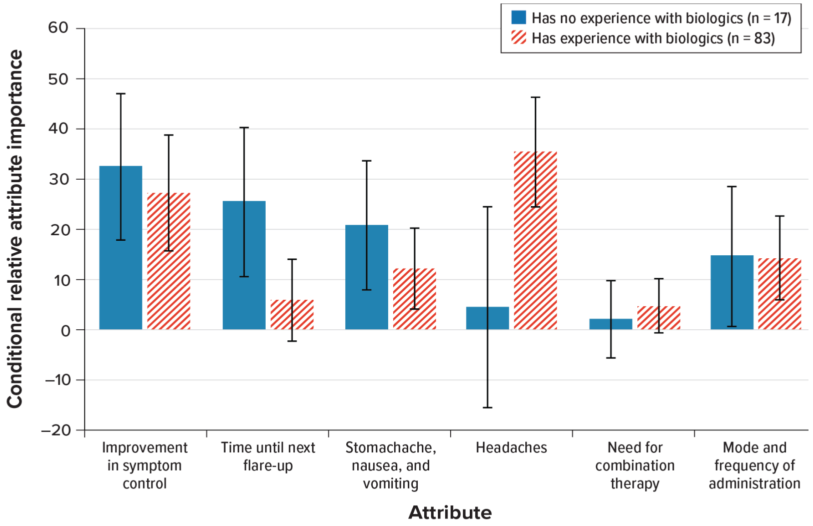

IV = intravenous.

Figure S22. Preference Weights and Conditional Relative Importance Estimates: Adolescents With Injection Experience Versus Adolescents With No Injection Experience

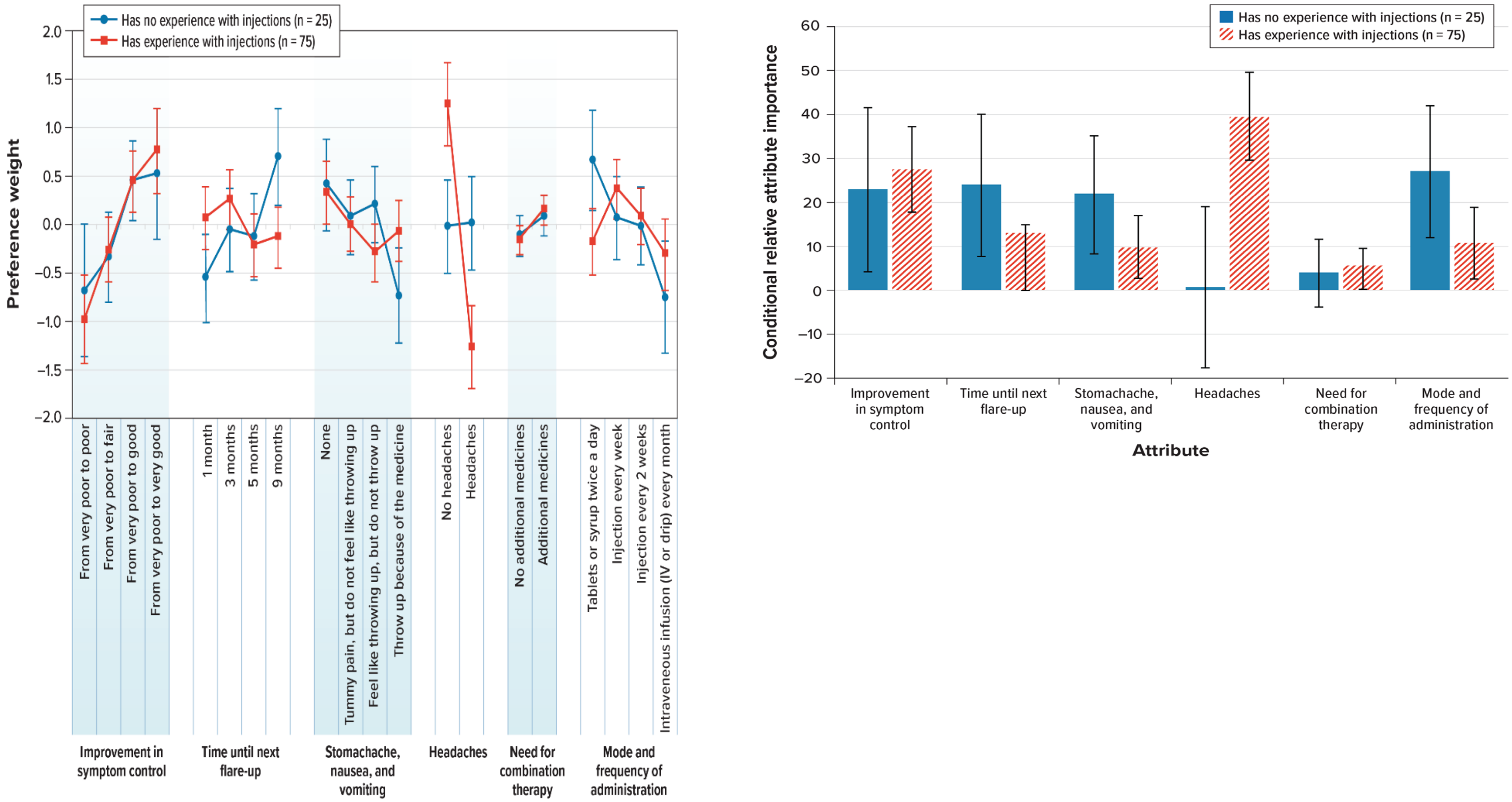

IV = intravenous.

Figure S23. Preference Weights and Conditional Relative Importance Estimates: Adolescents Who Have Experienced Headaches as a Side Effect of Their Treatment Versus Adolescents With No Experience With Headaches as a Side Effect of Their Treatment

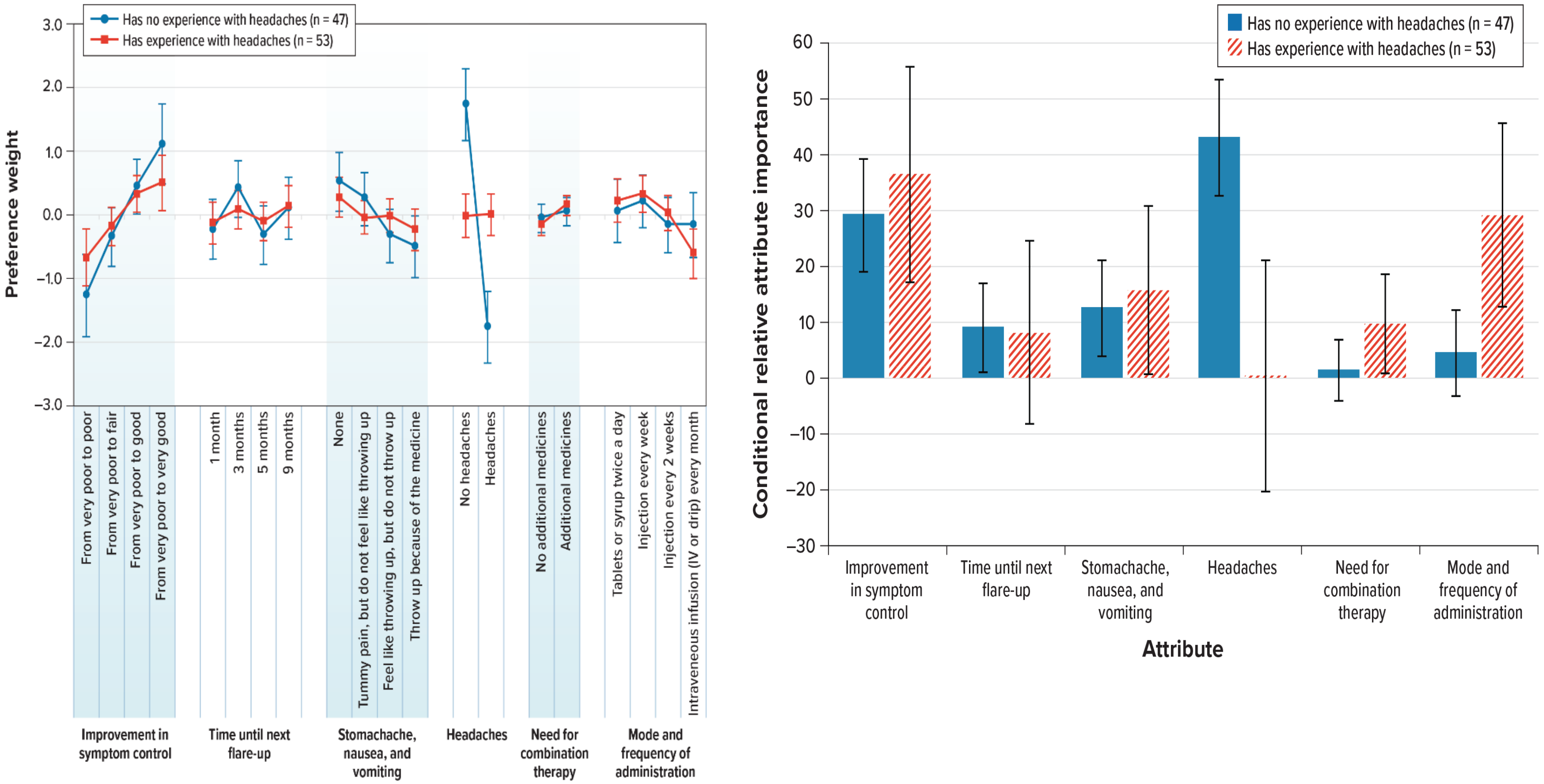

IV = intravenous.

Figure S24. Preference Weights and Conditional Relative Importance Estimates: Adolescents Who Have Experienced Stomachaches as a Side Effect of Their Treatment Versus Adolescents With No Experience With Stomachaches as a Side Effect of Their Treatment

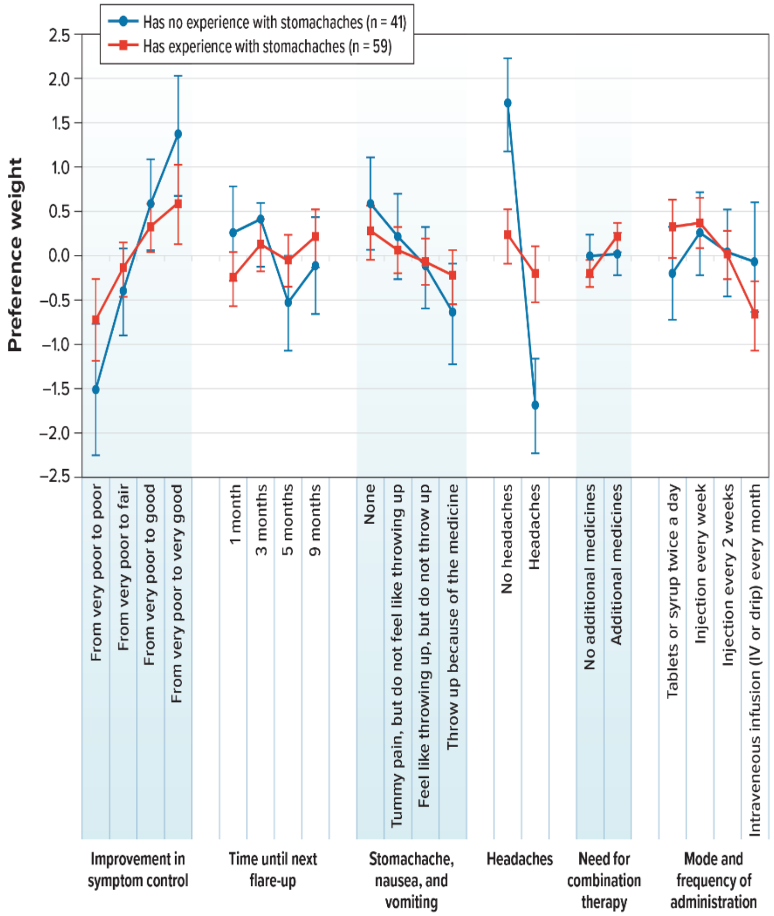

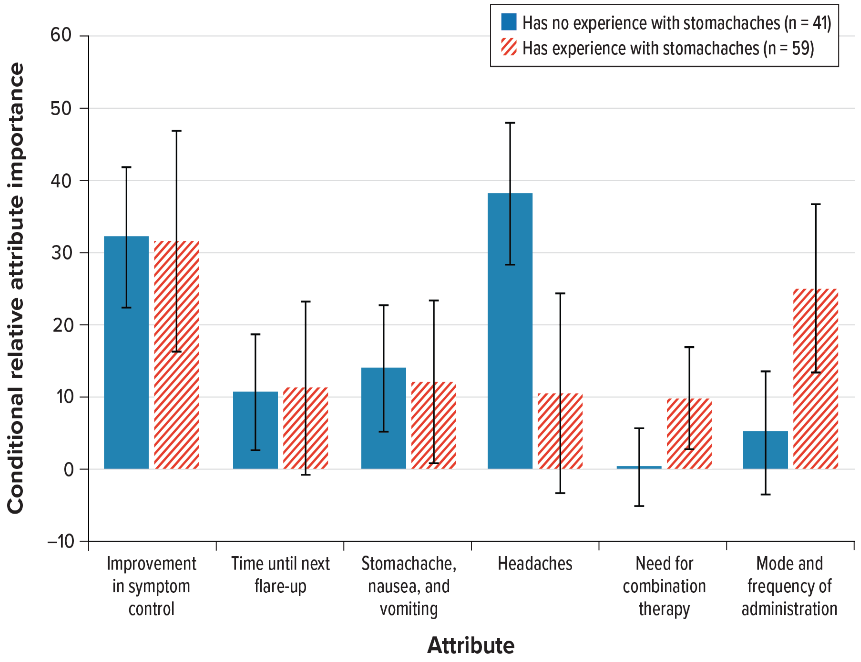

IV = intravenous.

Figure S25. Preference Weights and Conditional Relative Importance Estimates: Adolescents Who Have Experienced Vomiting as a Side Effect of Their Treatment Versus Adolescents With No Experience With Vomiting as a Side Effect of Their Treatment

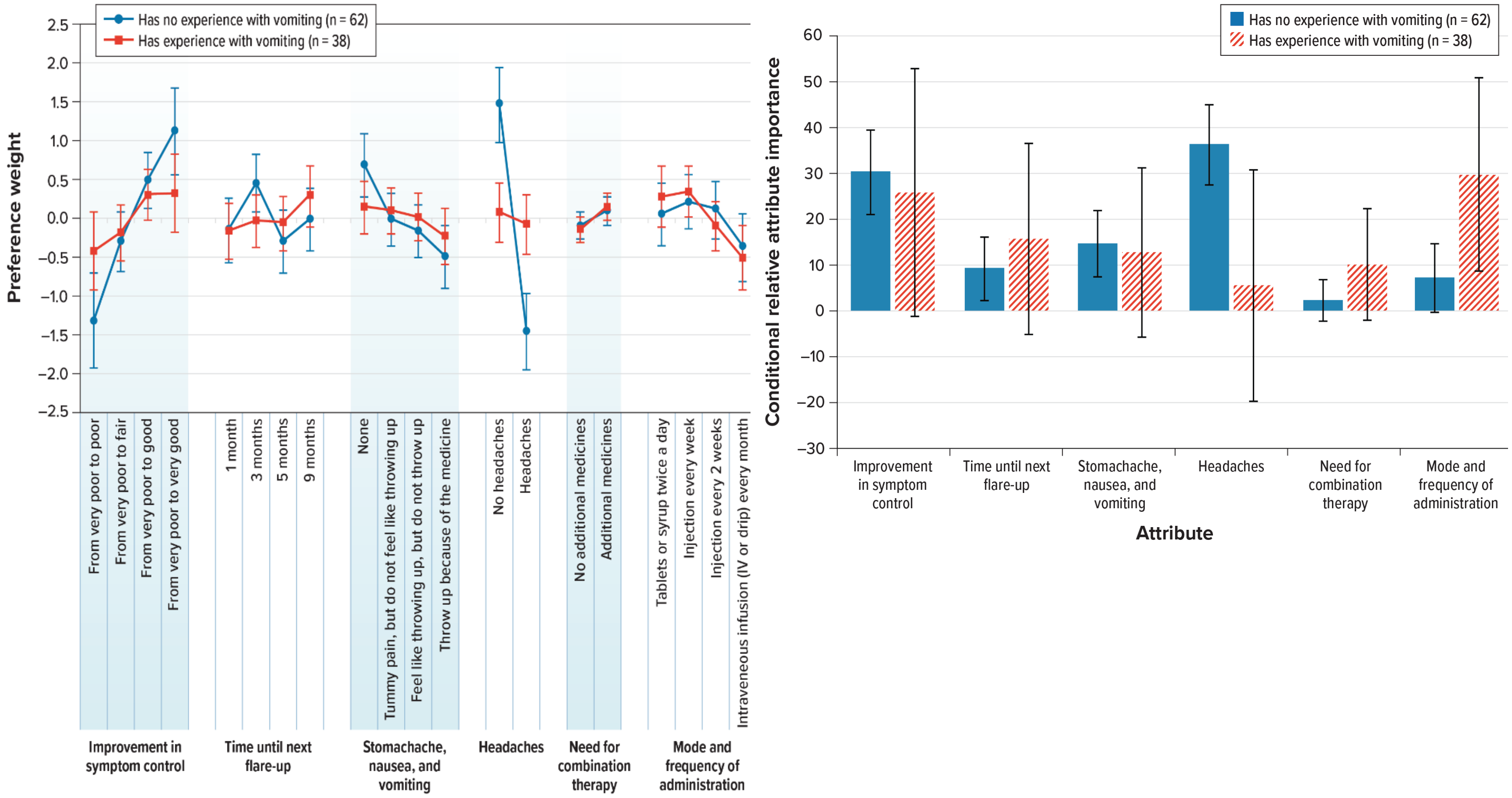

IV = intravenous.

Figure S26. Preference Weights and Conditional Relative Importance Estimates: Caregivers With a Child Younger Than Median Age Versus Caregivers With a Child at or Older Than Median Age

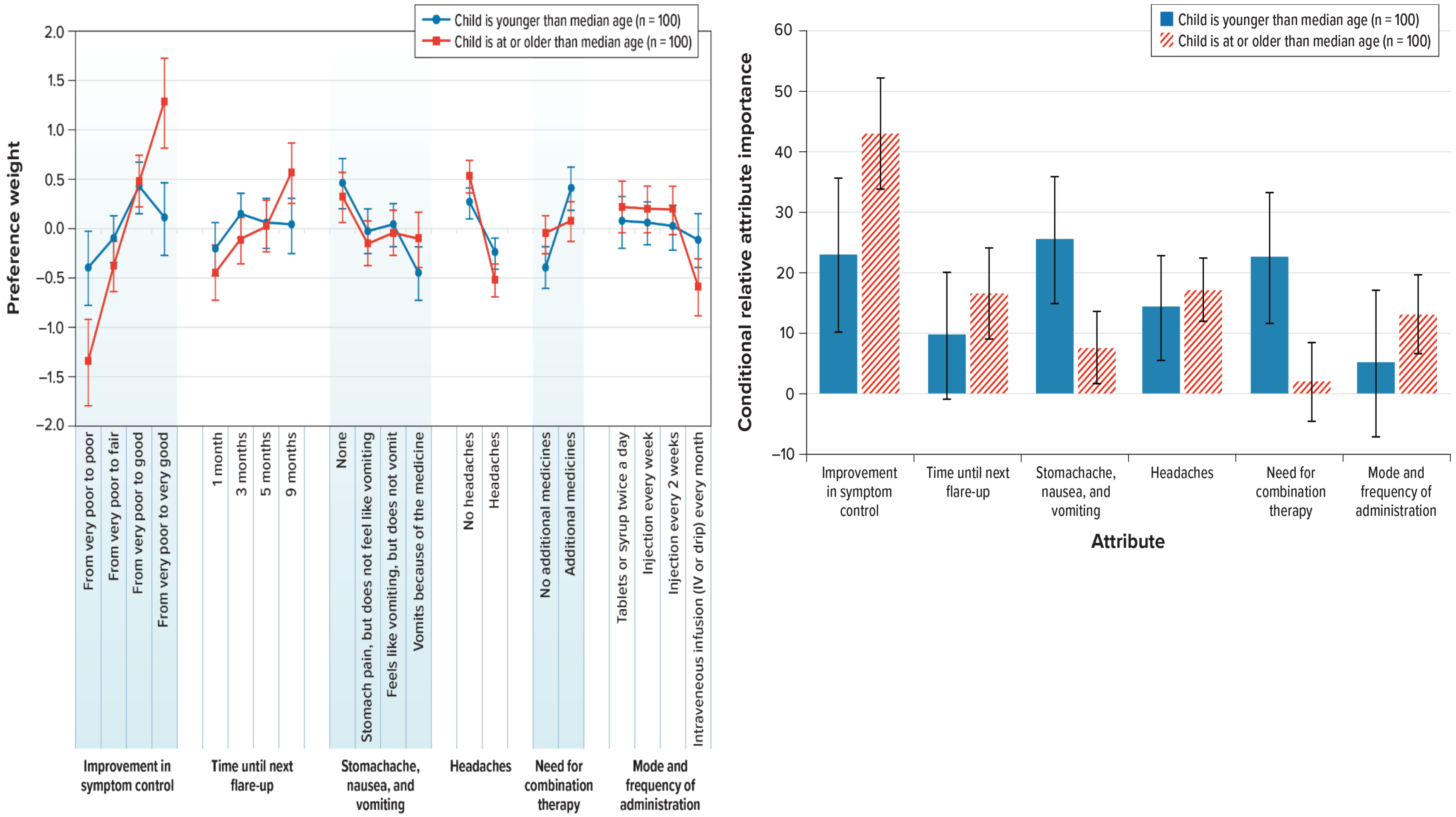

IV = intravenous.

Figure S27. Preference Weights and Conditional Relative Importance Estimates: Caregivers With a Child Who Identifies as Female Versus Caregivers With a Child Who Does Not Identify as Female

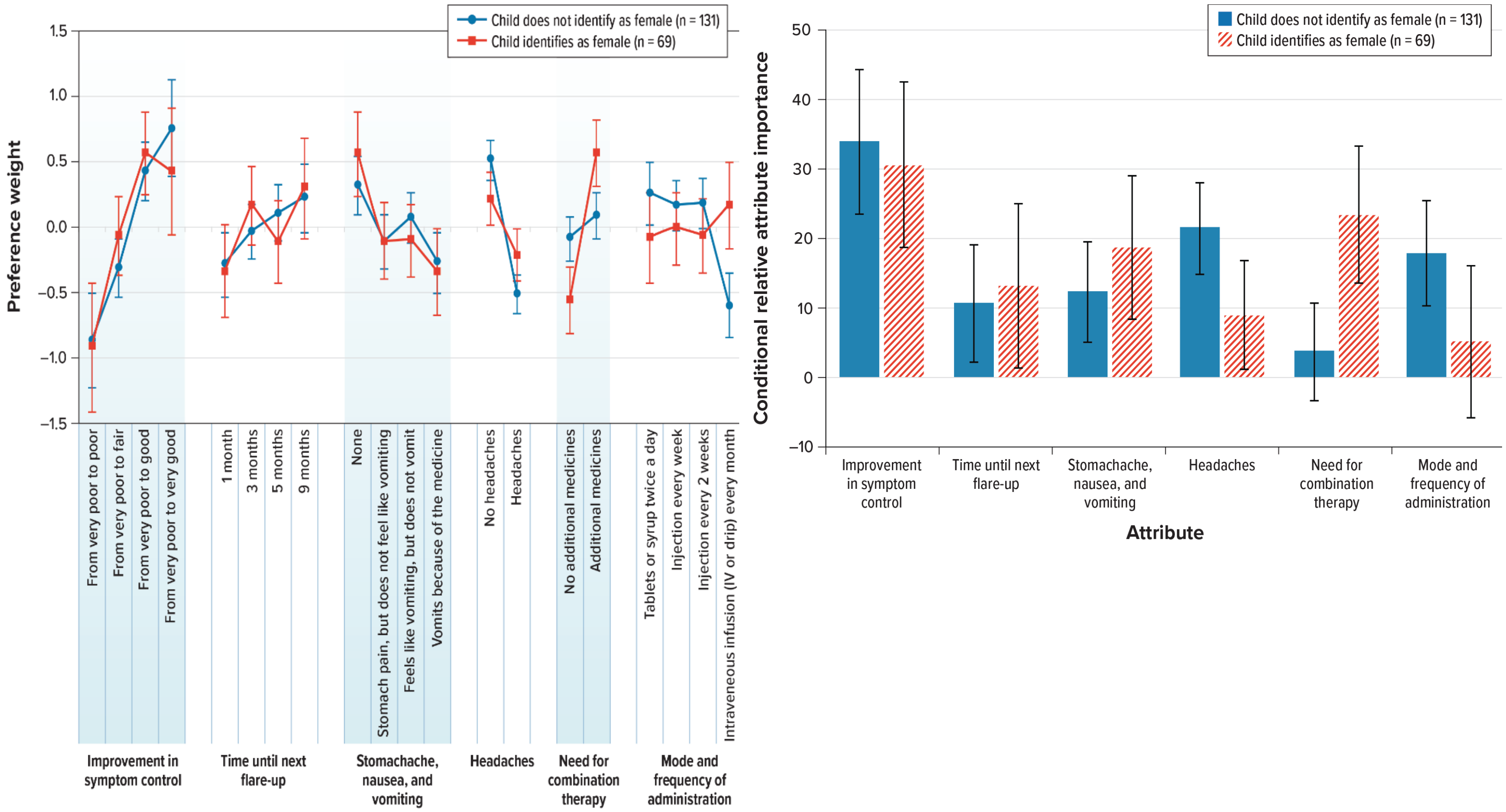

IV = intravenous.

Figure S28. Preference Weights and Conditional Relative Importance Estimates: Caregivers With a 4-Year Degree or Higher Versus Caregivers With Less Than a 4-Year Degree

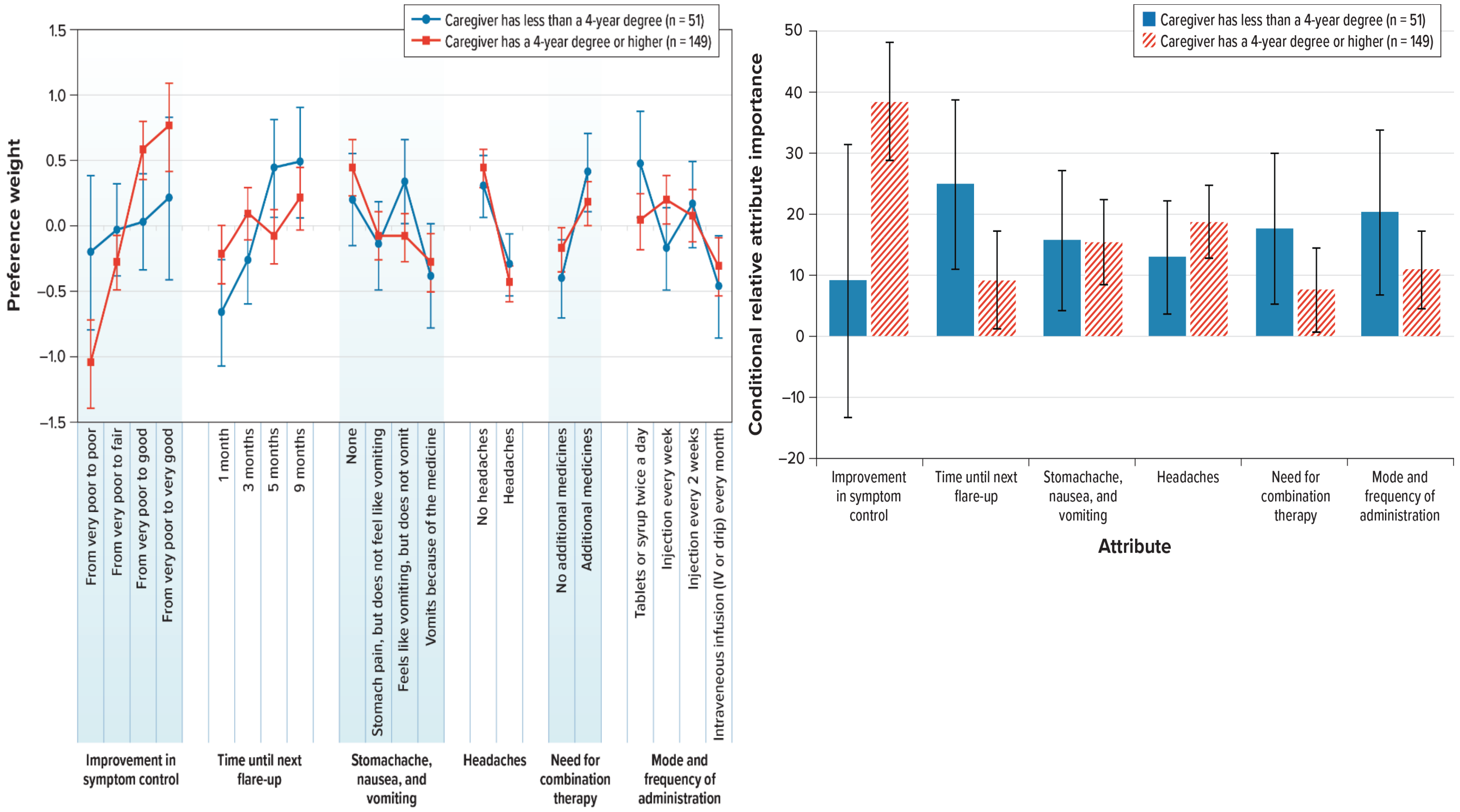

IV = intravenous.

Figure S29. Preference Weights and Conditional Relative Importance Estimates: Caregivers With a Child Who Has Experience With Methotrexate Versus Caregivers With a Child Who Has No Experience With Methotrexate

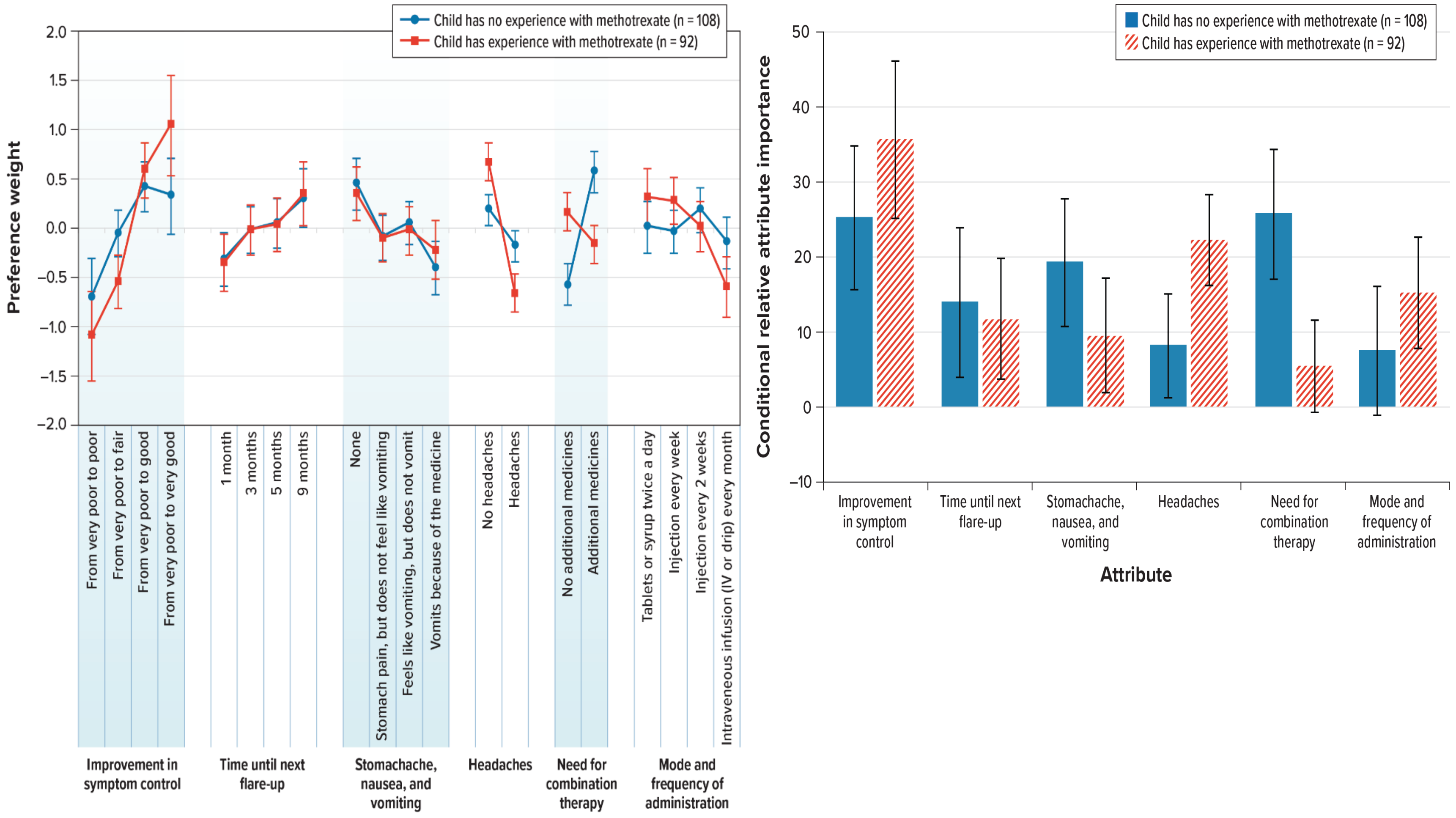

IV = intravenous.

Figure S30. Preference Weights and Conditional Relative Importance Estimates: Caregivers With a Child Who Has Experience With Biologics Versus Caregivers With a Child Who Has No Experience With Biologics

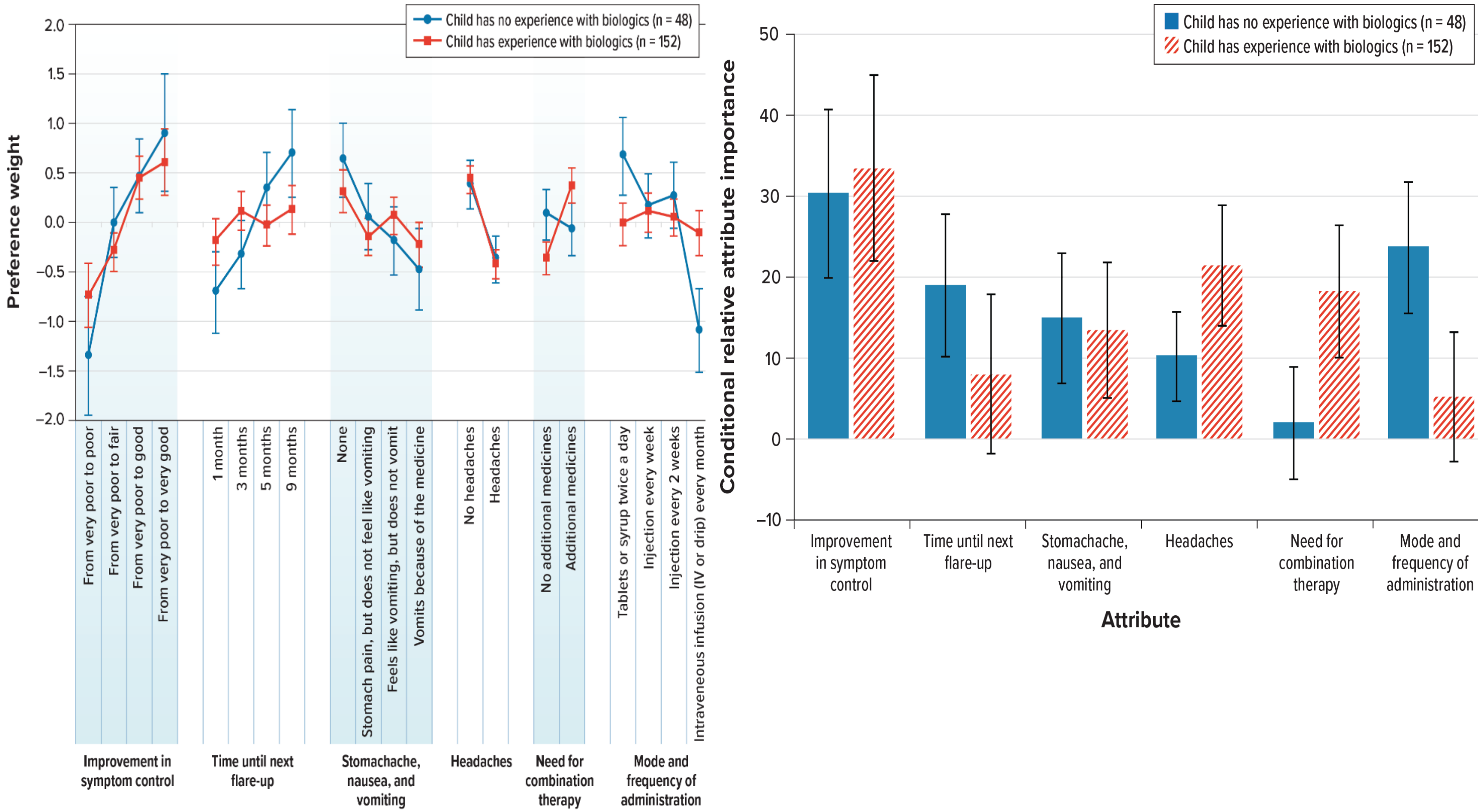

IV = intravenous.

Figure S31. Preference Weights and Conditional Relative Importance Estimates: Caregivers With a Child Who Has Experience With Injections Versus Caregivers With a Child Who Has No Experience With Injections

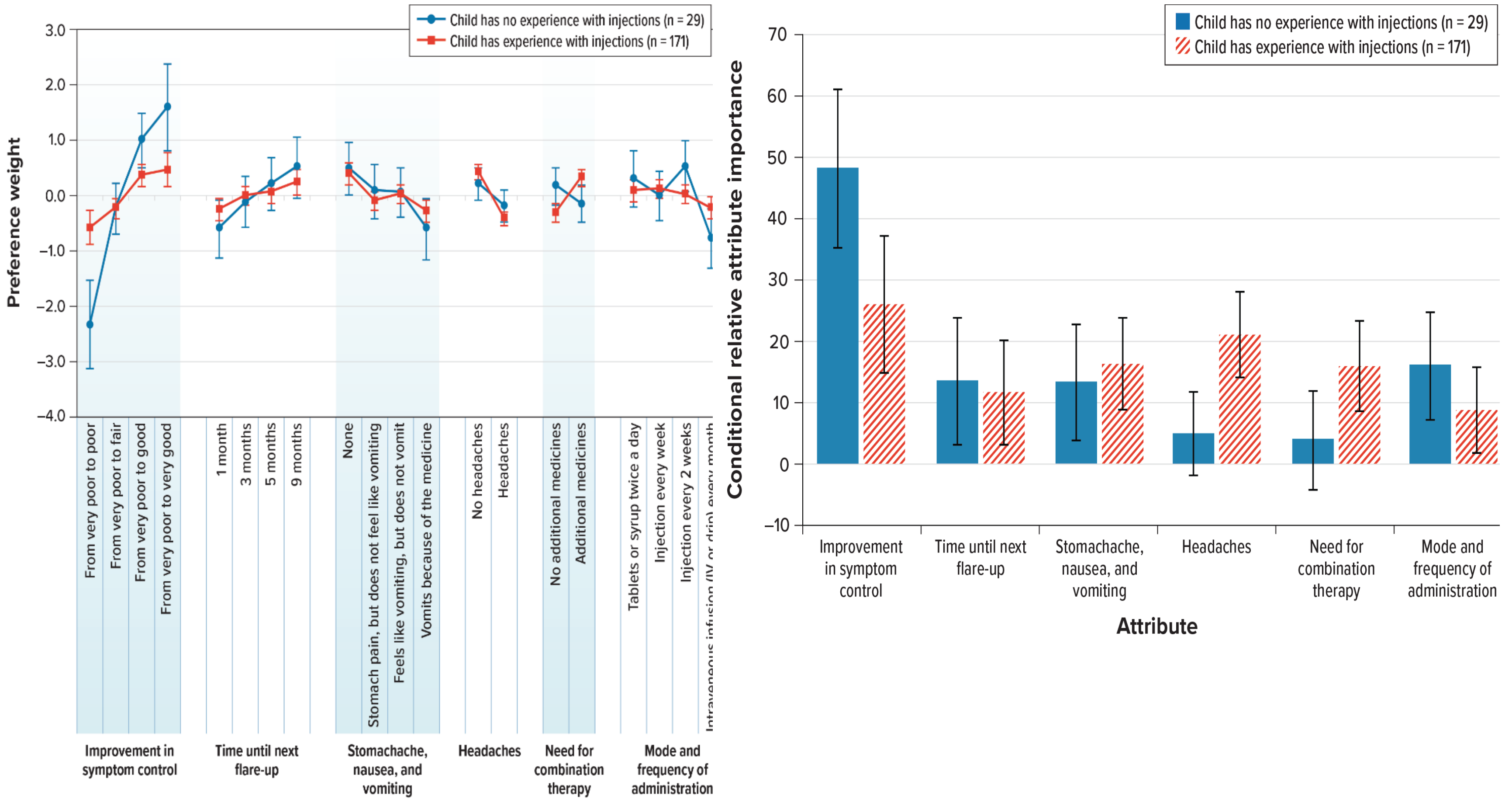

IV = intravenous.

Figure S32. Preference Weights and Conditional Relative Importance Estimates: Caregivers With a Child Who Has Experience With Headaches as a Side Effect of Their Treatment Versus Caregivers With a Child Who Has No Experience With Headaches as a Side Effect of Their Treatment

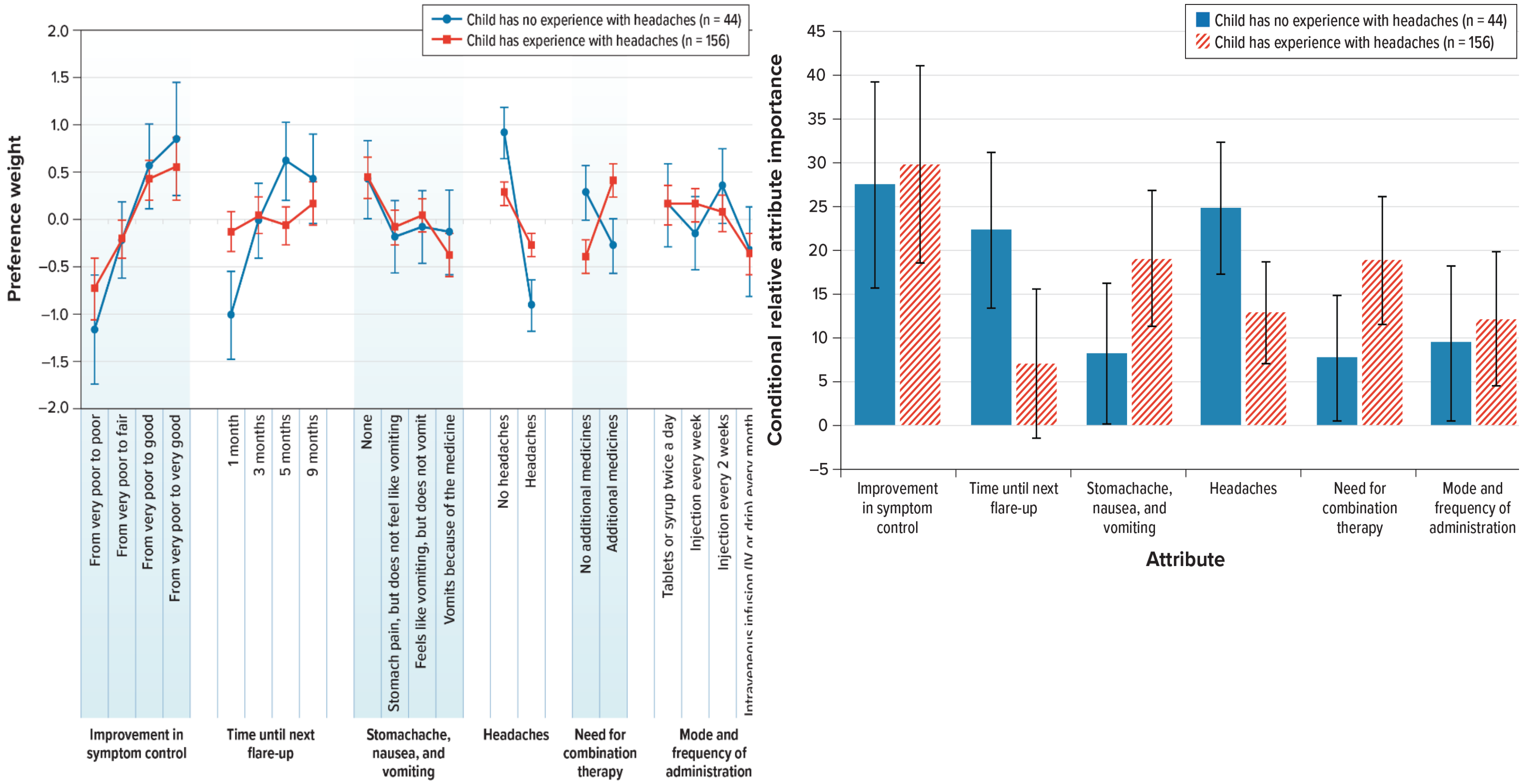

IV = intravenous.

Figure S33. Preference Weights and Conditional Relative Importance Estimates: Caregivers With a Child Who Has Experience With Stomachaches as a Side Effect of Their Treatment Versus Caregivers With a Child Who Has No Experience With Stomachaches as a Side Effect of Their Treatment

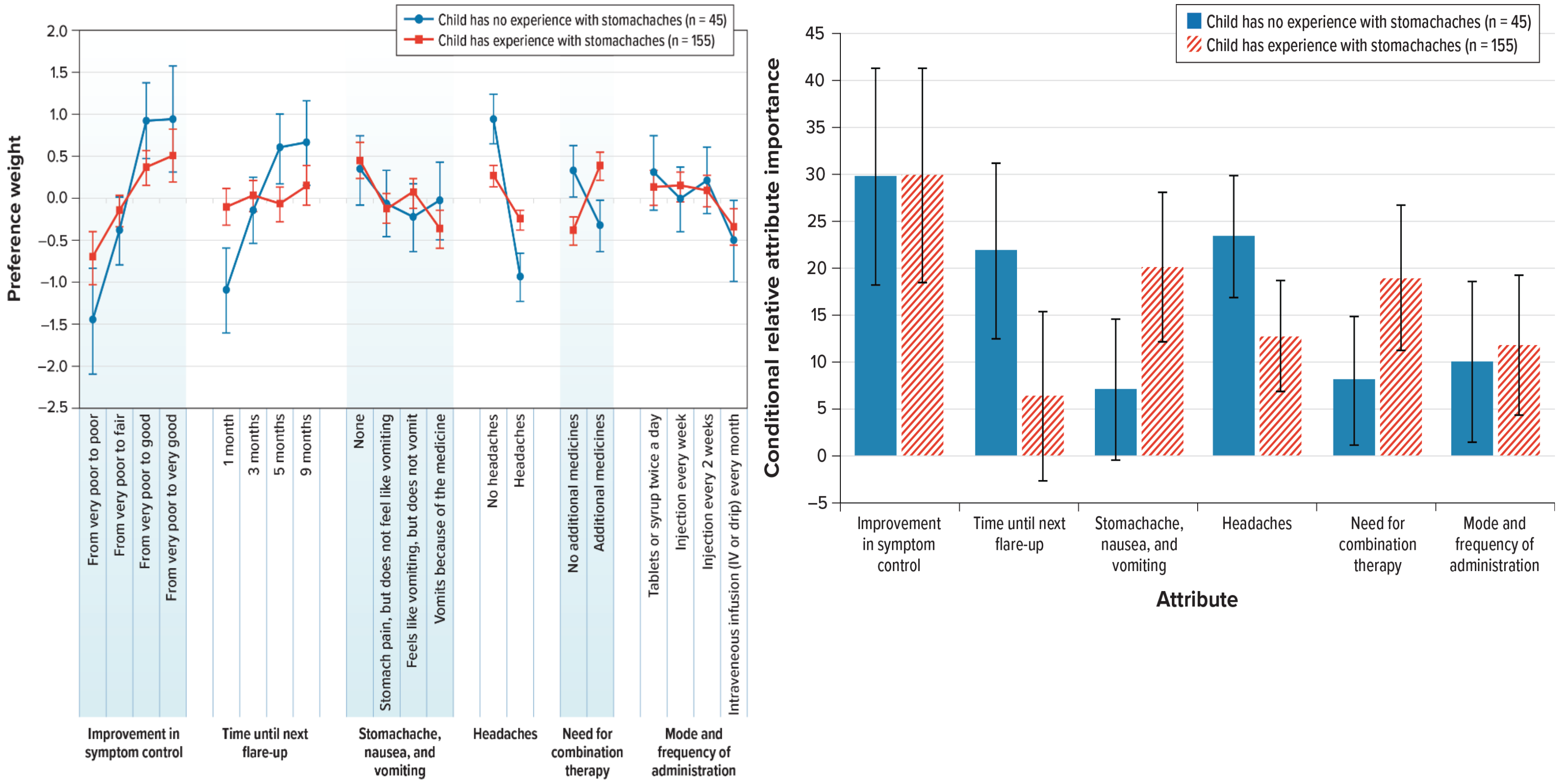

IV = intravenous.

Figure S34. Preference Weights and Conditional Relative Importance Estimates: Caregivers With a Child Who Has Experience With Vomiting as a Side Effect of Their Treatment Versus Caregivers With a Child Who Has No Experience With Vomiting as a Side Effect of Their Treatment

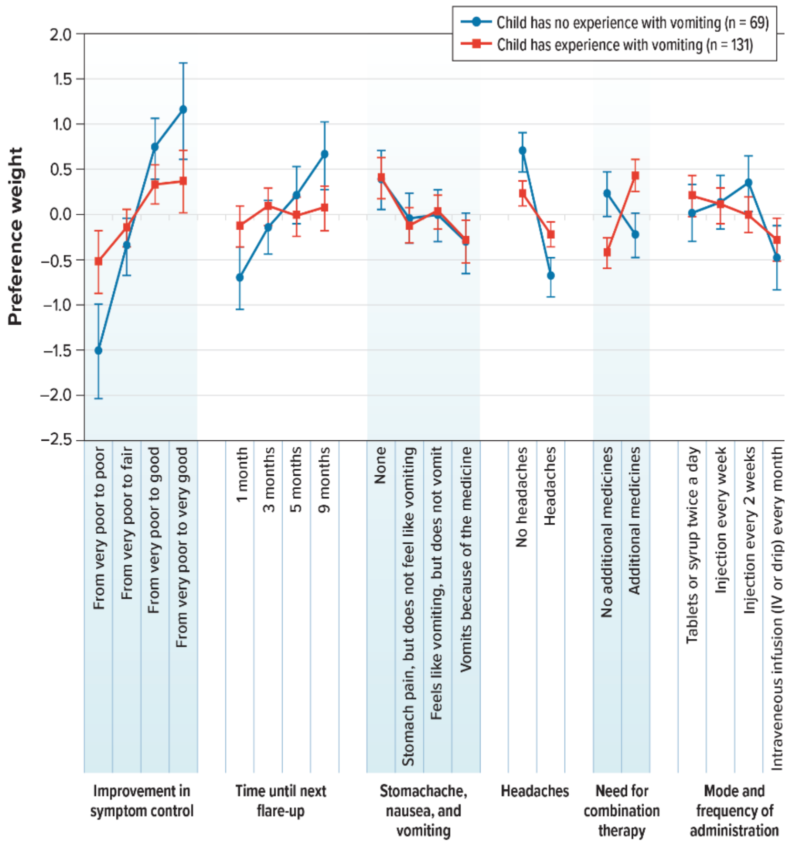

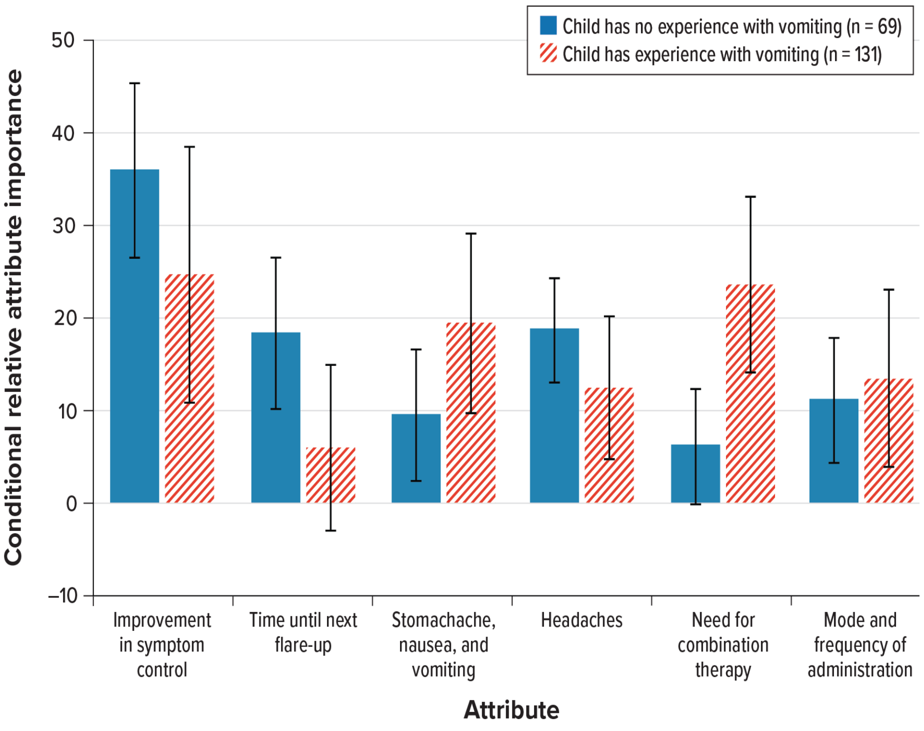

IV = intravenous.
